# A hunting ground for predatory bacteria at the Zhenbei seamount in the South China Sea

**DOI:** 10.1093/ismeco/ycaf042

**Published:** 2025-03-05

**Authors:** Zhimeng Li, Dayu Zou, Rulong Liu, Juntong Pan, Junkai Huang, Jun Ma, Liting Huang, Jiani He, Lulu Fu, Xiaowei Zheng, Minxiao Wang, Jiasong Fang, Hailiang Dong, Meng Li, Li Huang, Xin Dai

**Affiliations:** Southern Marine Science and Engineering Guangdong Laboratory (Guangzhou), No. 1119 Haibin Road, Guangzhou 511458, China; State Key Laboratory of Microbial Resources, Institute of Microbiology, Chinese Academy of Sciences, No. 1 West Beichen Road, Beijing 100101, China; Archaeal Biology Center, Synthetic Biology Research Center, Shenzhen Key Laboratory of Marine Microbiome Engineering, Key Laboratory of Marine Microbiome Engineering of Guangdong Higher Education Institutes, Institute for Advanced Study, Shenzhen University, 3688 Nanhai Avenue, Shenzhen 518060, China; College of Oceanography and Ecological Science, Shanghai Ocean University, No. 999 Huchenghuan Road, Shanghai 201306, China; State Key Laboratory of Microbial Resources, Institute of Microbiology, Chinese Academy of Sciences, No. 1 West Beichen Road, Beijing 100101, China; Center for Geomicrobiology and Biogeochemistry Research, State Key Laboratory of Geomicrobiology and Environmental Changes, China University of Geosciences, No. 29 Xueyuan Road, Beijing 100083, China; Southern Marine Science and Engineering Guangdong Laboratory (Guangzhou), No. 1119 Haibin Road, Guangzhou 511458, China; State Key Laboratory of Microbial Resources, Institute of Microbiology, Chinese Academy of Sciences, No. 1 West Beichen Road, Beijing 100101, China; CAS Key Laboratory of Marine Ecology and Environmental Sciences, Institute of Oceanology, Chinese Academy of Sciences, 7 Nanhai Road, Qingdao 266071, China; College of Oceanography and Ecological Science, Shanghai Ocean University, No. 999 Huchenghuan Road, Shanghai 201306, China; College of Oceanography and Ecological Science, Shanghai Ocean University, No. 999 Huchenghuan Road, Shanghai 201306, China; Center of Deep-Sea Research, Institute of Oceanology, Chinese Academy of Sciences, 7 Nanhai Road, Qingdao 266071, China; State Key Laboratory of Microbial Resources, Institute of Microbiology, Chinese Academy of Sciences, No. 1 West Beichen Road, Beijing 100101, China; Center of Deep-Sea Research, Institute of Oceanology, Chinese Academy of Sciences, 7 Nanhai Road, Qingdao 266071, China; College of Oceanography and Ecological Science, Shanghai Ocean University, No. 999 Huchenghuan Road, Shanghai 201306, China; Center for Geomicrobiology and Biogeochemistry Research, State Key Laboratory of Geomicrobiology and Environmental Changes, China University of Geosciences, No. 29 Xueyuan Road, Beijing 100083, China; Archaeal Biology Center, Synthetic Biology Research Center, Shenzhen Key Laboratory of Marine Microbiome Engineering, Key Laboratory of Marine Microbiome Engineering of Guangdong Higher Education Institutes, Institute for Advanced Study, Shenzhen University, 3688 Nanhai Avenue, Shenzhen 518060, China; Southern Marine Science and Engineering Guangdong Laboratory (Guangzhou), No. 1119 Haibin Road, Guangzhou 511458, China; State Key Laboratory of Microbial Resources, Institute of Microbiology, Chinese Academy of Sciences, No. 1 West Beichen Road, Beijing 100101, China; College of Life Sciences, University of Chinese Academy of Sciences, No. 1 Yanqihu East Rd, Beijing 100049, China; State Key Laboratory of Microbial Resources, Institute of Microbiology, Chinese Academy of Sciences, No. 1 West Beichen Road, Beijing 100101, China; College of Life Sciences, University of Chinese Academy of Sciences, No. 1 Yanqihu East Rd, Beijing 100049, China

**Keywords:** seamounts, microbial communities, metagenomics, metatranscriptomics, *Myxococcota*, *Bdellovibrionota*, predatory bacteria

## Abstract

Seamounts are critical marine biodiversity hot spots, while the metabolic activity of their microbial community remains largely unknown. In this study, we investigated the diversity and activity of free-living and particle-attached microorganisms in the surface, middle, and bottom layers of seawater at the Zhenbei seamount in the South China Sea using omics approaches, including 16S ribosomal RNA (rRNA)/16S rDNA ratio analysis. Over 20 phyla were detected, with *Proteobacteria*, *Actinobacteriota*, *Cyanobacteria*, *Bacteroidota*, *Thaumarchaeota*, and *Planctomycetota* being predominant. Surprisingly, *Bdellovibrionota* and *Myxococcota*, the two well-known predatory bacteria, exhibited exceptionally higher rRNA/rDNA ratios than the other phyla, with rRNA abundances being 10- or even 200-fold higher than their rDNA abundances. These metabolically active predatory bacteria are mainly uncultured species. A total of 23 *Myxococcota* metagenome-assembled genomes (MAGs) and 12 *Bdellovibrionota* MAGs were assembled. The most highly overexpressed genes frequently detected in these MAGs were those that encode flagellum and pilus proteins as well as T4-like virus tail tube protein, indicating that these predator bacteria were likely active in hunting. Our results suggest that seamounts may serve as hunting grounds for predatory bacteria, which may be involved in controlling the flows of elements and energy in the seamount microbial communities and, thus, in shaping the seamount ecosystems.

## Introduction

Seamounts, isolated or clustered, are subaquatic mountains that rise at least 1000 m above the surrounding seafloor [1, 2]. Current estimates of the number of seamounts (ranged from >150 000 to >25 million globally) indicate that they cover a large area of the deep seabed and host one of the major biomes of the ocean. However, only a tiny fraction (<0.002%) of the seamounts across the global ocean have been sampled [3, 4]. Changes in physical oceanographic conditions at seamounts, such as enhanced vertical mixing, Taylor columns/cones, internal waves, current acceleration, and mesoscale ocean eddies, lead to the enrichment of particulate organic matter (POM) and inorganic nutrients, making seamounts unique ecosystems, or “hotspots,” for marine megafauna, such as cetaceans, pinnipeds, seabirds, sharks, tuna, billfish, and other oceanic pelagic fish [4–11]. Over the years, the ecology of metazoans, including zooplankton, micronekton, and fish at seamounts, has received considerable attention [12, 13].

Microbial communities are a key driver of biogeochemical cycles in the oceans and respond quickly and specifically to environmental disturbances [14]. As an attractive area for large predators to forage, seamounts are conceivably places where microorganisms are abundant and active. However, our understanding of microbial processes in seamount ecosystems remains rudimentary [3, 6, 15–21]. Only a limited number of studies have been carried out to investigate the diversity and ecology of microbial communities in water and sediments of seamounts. These studies have revealed the high richness, diversity, and spatial distribution heterogeneity of microbial communities (β-diversity) in seamounts [3, 15, 19, 20, 22]. Surveys based on the 16S ribosomal RNA (rRNA) gene (rDNA) frequently show that *Proteobacteria*, *Bacteroidetes*, *Actinobacteriota, Planctomycetota*, *Cyanobacteria*, as well as *Thaumarchaeota*, *Chloroflexi*, *Acidobacterota*, *Verrucomicrobia*, *Marinimicrobia* (SAR406 clade), SAR324 clade, are dominant phyla in seamounts [3, 15, 19, 20]. Since these 16S rDNA-based studies do not distinguish between active, dormant, and dead cells [23–28], the microbial processes of seamounts are still elusive.

Unlike rDNA, ribosomal RNA (rRNA) has been shown to be positively correlated with the growth rate of many bacteria [29–31]. Therefore, rRNA-based approaches have been used to monitor active and living populations in diverse habitats [32, 33]. Recently, rRNA/rDNA has increasingly been used as a proxy for microbial activity to quantify taxon-specific activities in a microbial community and to identify clades for which there is uncoupling between specific activity and abundance in marine habitats [25, 33–36]. It is generally believed that mean rRNA/rDNA ratios for most operational taxonomic units (OTUs) are close to or equal to 1.0, and strong correlations between rRNA and rDNA frequencies have also been observed among different taxa [25, 37–39]. As revealed by the rRNA/rDNA measurements, low-abundance microbes might be disproportionately active in some habitats, whereas some of the most abundant microbes might exhibit low metabolic activity [25, 33].

The South China Sea (SCS) is one of the largest semienclosed marginal seas in the Western Pacific Ocean with complex physical and chemical gradients over spatial scales. In this study, we investigated microbial communities at the Zhenbei seamount, one of the major components of the Zhenbei–Huangyan seamount chain located at the center of the SCS [18, 40]. Many seamounts are scattered across the vast abyssal plain of the SCS, causing the “seamount effects” and fundamentally affecting primary production and the community structures of phytoplankton and sedimentary microorganisms [3, 16, 18, 22, 41]. Samples taken at various depths were subjected to multi-omics analyses, including the determination of the rRNA/rDNA abundance ratio. We show that microbes belonging to phyla *Bdellovibrionota* and *Myxococcota*, two well-known predatory bacteria, were strikingly more active than those in other phyla in these samples. These findings are consistent with the notion that bacterial predation may occur extensively, driving rapid turnover of microbial cells in seamount ecosystems.

## Materials and methods

### Samples and parameters

**Figure 1:**
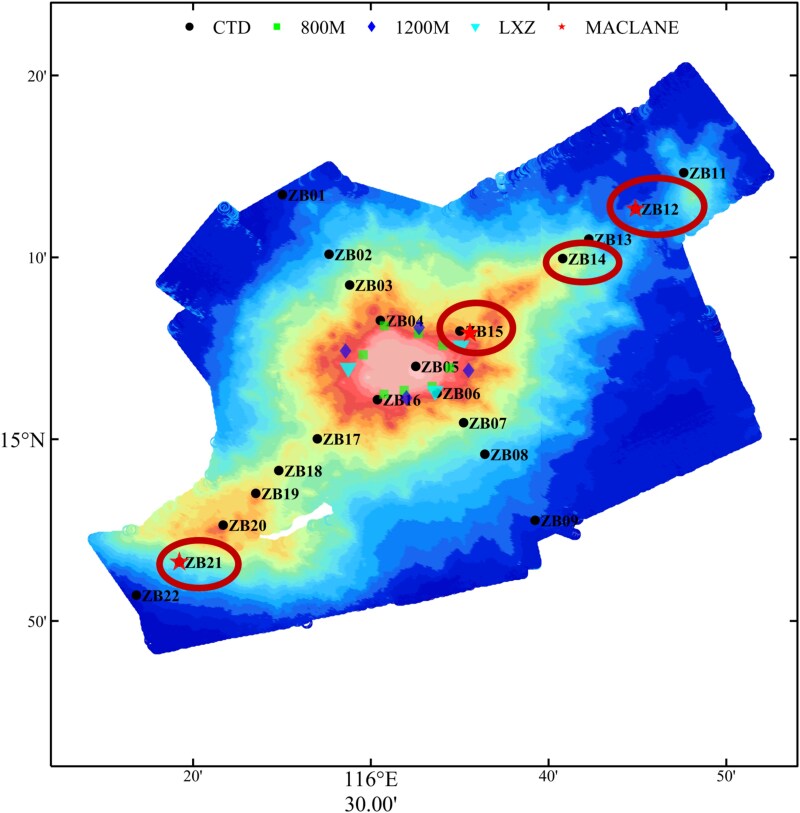
Sampling sites. Locations of the sampling sites in the Zhenbei seamount are circled in the figure.

Twenty-four seawater samples (100 l each) were taken in July 2022 at four stations around the Zhenbei seamount from the surface (5–50 m), middle, and bottom layers in the SCS during the “KEXUE” expedition cruise (Fig. 1; Supplementary Table S1), and named according to the sampling site and depth. The samples were immediately filtered sequentially through 3.0- and 0.2-μm polycarbonate filters (PC, Millipore) to collect particle-attached (PA) and free-living (FL) microbes, respectively, using a multiunit large volume in-situ filtration system (MULVFS) fitted with battery-operated McLane (WTS-LV, American) in-situ pumps. Each filter was divided into two parts, with one part immersed in RNAsafer II Reagent (15 ml; OMEGA bio-tek, R0424-02). The two parts were stored separately at −80°C.

Physical characteristics of the water samples, including depth, salinity, and temperature, were measured with CTD sensors during sampling. Concentrations of dissolved oxygen (DO) were determined according to the Winkler method [42, 43]. Briefly, an aliquot (50 ml) of the seawater sample was added to an iodine flask, to which alkaline potassium iodide and manganese sulfate were added. The sample was titrated with a standard solution of sodium thiosulfate (0.01 mM) The pH values of the seawater samples were measured using a pH meter (Thermo Scientific Orion 5-star, USA) [42]. Concentrations of seawater nutrients, including nitrate (NO_3_-N), nitrite (NO_2_-N), ammonium (NH_4_-N), phosphate (PO_4_-P), and silicate (SiO_3_-Si), were determined using a nutrient automatic analyzer (SEAL QuAAtro) [44]. Concentrations of POC and PON were determined with an elemental analyzer (Thermo Fisher Scientific Flash EA 1112, USA) [45].

### DNA/RNA preparation, PCR, and sequencing

RNA and DNA in microorganisms collected on the filters were extracted with the RNeasy® PowerSoil® Total RNA Kit and the RNeasy® PowerSoil® DNA Elution Kit (Qiagen, Germany) according to the manufacturer’s protocols. Due to technical difficulties presumably associated with low biomass, we were unable to obtain sequence data from some of the samples (Supplementary Table S2). The concentration and quality of final DNA preparations were determined by NanoDrop 2000 UV–vis spectrophotometer (Thermo Scientific, Wilmington, DE, USA). DNA samples were amplified with the barcoded primers 515F (5′-GTGCCAGCMGCCGCGG-3′) and 907R (5′-CCGTCAATTCMTTTRAGTTT-3′) targeting the V4–V5 hypervariable regions of the 16s rRNA gene. RNAs were also amplified with the same primers after reverse transcription into cDNA with the Promega GoScript™ Reverse Transcription System (Promega, USA). Polymerase chain reaction (PCR) reactions and thermocycler program followed Liu *et al.* [25]. Each PCR reaction (50 μl) carried out in triplicates.

Equimolar amplicons were pooled and paired-end sequenced (2 × 250) on an Illumina MiSeq platform (Illumina, San Diego, CA, USA) according to the standard protocols of Majorbio Bio-Pharm Technology Co. Ltd. (Shanghai, China). The metagenomic and metatranscriptomic sequencing were performed at Guangdong Magigene Biotechnology Co., Ltd. (Guangzhou, China) using standard protocols.

### rDNA and rRNA amplicon data processing

A total of 26 raw amplicon sequencing data, including 14 rDNA data and 12 rRNA data, were obtained in this study. All data were processed via the QIIME2 platform (v2022.2) [46]. First, the primer sequence was removed from the raw sequences using Cutadapt (v1.9.1) [47], and the sequences were then imported into the QIIME2 platform using “qiime tools import”. The quality filtering was carried out using “qiime quality-filter q-score” with p-min-quality set at 20 and p-min-length-fraction at 0.85. Second, DADA2 was employed to denoise, remove chimera, and generate features using “qiime dada2 denoise-paired” with the p-min-overlap set at 12 [48]. Features were clustered at 97% sequence identity using “qiime vsearch cluster-features-de-novo” in the vsearch module to align the representative OTUs. Taxonomic information for each representative OTU was assigned in the feature-classifier module using “qiime feature-classifier classify-sklearn” according to the SILVA Nr99 database (v138). The sequencing data of each sample was normalized at the same sequencing depth (i.e. 20 818 bp, the sequencing data of ZB15-BPA, which represented the least amount of data among all samples), and subjected to alpha (i.e. OTU richness and Shannon) and beta diversity (i.e. the Bray–Curtis dissimilarity matrix) analyses using “qiime diversity core-metrics-phylogenetic.”

Samples were clustered using the unweighted pair-group method with arithmetic (UPGMA) based on the Bray–Curtis dissimilarity matrix of the microbial community composition of rDNA data. Analysis of variance (ANOVA) followed by Tukey’s honestly test was employed to compare the average community composition of rDNA samples between different biomes (i.e. FL and PA) and different water depth (i.e. S, M, and B). Besides, the relative abundance of *Bdellovibrionota* and *Myxococcota* and the alpha diversity indices of the rDNA and rRNA samples were compared as well. Principal coordinate analysis (PCoA) and permutational multivariate analysis of variance (PERMANOVA) were used to depict the prokaryotic community composition shift between different samples based on the Bray–Curtis dissimilarity matrices on the OTU level. The above analyses were conducted using the vegdist and anosim function in the vegan package in R (v3.6, R Development Core Team, Vienna, Austria). The phylogenetic analysis of OTUs belonging to phyla *Bdellovibrionota* and *Myxococcota* was conducted on the QIIME2 platform. Representative sequences were aligned using “qiime alignment mafft,” and the phylogenetic tree was built using “qiime phylogeny fasttree” and visualized using the online platform iTOL [49].

### Metagenomic assembly, binning, and omics analysis

Raw metagenomic reads were trimmed using the read_qc module within MetaWRAP (v1.3.2) [50]. Trimmed reads from each sample were assembled using MEGAHIT (v1.2.9) with the “meta-large” preset parameter [51]. Scaffolds longer than 2000 bp generated from the assembly were binned into draft genomes using MetaBAT2 (v2.12.1) with six sets of parameters [52]. These six sets of bins were then refined using the bin_refinement module in MetaWRAP to obtain optimized metagenome-assembled genomes (MAGs). The quality assessment of all MAGs, including completeness, contamination, and heterogeneity, was conducted using CheckM (v1.0.12) [53]. MAGs with completeness greater than 50% and contamination <10% were used in downstream analysis. Additionally, genomes were dereplicated at 95% average nucleotide identity (species level) using dRep (v3.4.5) [54]. Taxonomic classification of the MAGs was performed using GTDB-Tk (v2.3.2) [55].

Gene prediction was carried out using Prodigal (v2.6.3) with the “-p meta” for all dereplicated MAGs to identify open reading frames (ORFs). These ORFs were then annotated by eggNOG-mapper [56]. METABOLIC software was used to predict the metabolic and biogeochemical trait profiles of the *Bdellovibrionota* and *Myxococcota* MAGs [57]. The predicted metabolic capabilities, the presence and absence of metabolic functions, and the contribution to various biogeochemical processes of each MAG were revealed using the “METABOLIC-C” module with default parameters. The CAZy numbers and hits, as well as the MEROPS peptidase numbers and hits for each MAG, were searched. AntiSMASH (v6.0) [58] was used with default parameters to predict biosynthetic gene clusters (BGCs) in the genomes.

The raw reads generated after metatranscriptomic sequencing were trimmed using Sickle and were then filtered with SortMeRna [59] with default settings to remove tRNA and rRNA sequences. The genomic and transcriptomic reads from each sample were separately mapped onto the dereplicated *Bdellovibrionota* and *Myxococcota* MAGs using CoverM (v0.6.1; https://github.com/wwood/coverm) (with -p bwa-mem, --min-read-percent-identity 95, --min-read-aligned-percent 50, and --min-covered-fraction 10) for the following abundance calculations. The Reads Per Kilobase per Million mapped reads (RPKM) and transcripts per million (TPM) values of all dereplicated MAGs were calculated to represent their relative abundance and transcript activity, respectively. The relative genomic and transcriptomic abundance of each MAG in a sample was calculated as the percentage of its value to the summed RPKM or TPM values of all MAGs. Besides, for each *Bdellovibrionota* or *Myxococcota* MAG, the TPM values of all ORFs in each sample were calculated as well.

### Phylogenetic and network analyses

A total of 35 representative *Bdellovibrionota* and *Myxococcota* MAGs were collected for the phylogenomic tree reconstruction. Multiple sequence alignments (MSA) of 120 concatenated conserved bacterial marker genes were retrieved from GTDB-Tk. The MSA data was used to generate phylogeny by applying IQ-TREE (v1.6.12) [60] with the mixture model of LG + F + R4 and with 1000 ultrafast bootstrapping, followed by rooting with MAD [61]. The best model was determined by ModelFinder [62], which is well supported by Bayesian Information Criterion. The phylogenomic tree was visualized using the online platform iTOL [49].

## Results

### Microbial diversity and activity at the Zhenbei seamount

Geochemical parameters, such as temperature, salinity, DO concentration, pH, and inorganic nutrients, were measured for samples collected from four sites around the Zhenbei seamount in the SCS, i.e. ZB12 (3523 m), ZB14 (2099 m), ZB15 (1092 m) and ZB21 (3087 m) (Fig. 1; Supplementary Fig. S1; Supplementary Table S1). Overall, these parameters and their variations with depth at the four sampling sites were similar. Total DNA and RNA were extracted from each sample collected on the filter. In general, we were more successful in obtaining various sequence data from ZB15 samples at all three depths or from the top water layer at all sites. Although the varied success in obtaining sequencing data for different samples prevented a systematic study of the microbial diversity, distribution, and connectivity along the horizontal and vertical gradients at the seamount, an analysis of active microbes in the seamount microbial community was possible because of the availability of both 16S rDNA (rDNA) and 16S rRNA (rRNA) sequencing datasets for a total of 12 samples. Moreover, since we obtained 26 FL and 19 PA datasets (Supplementary Table S2), a comparison between the FL and PA microbes in seamount was also possible.

After processing and resampling the rDNA and rRNA amplicon data (Supplementary Table S3), a total of 9486 OTUs were retrieved from 1 976 751 sequences (~76 000 sequences/sample), of which 1 879 641 sequences (95.1%) were assigned to Bacteria and 97 110 sequences (4.9%) to Archaea (Table 1; Supplementary Table S4). Among them, 6870 OTUs retrieved from 1 087 156 rDNA sequences (92.6% for Bacteria, 7.4% for Archaea) were obtained from 14 samples, and 4087 OTUs retrieved from 889 595 rRNA sequences (98.1% for Bacteria, 1.9% for Archaea) from 12 samples (Table 1). A comparison of alpha diversity showed that 10 out of 12 samples, from which both rDNA and rRNA were obtained, had higher Shannon indexes in rRNA than in rDNA (Supplementary Fig. S2 and Supplementary Table S5), suggesting the presence of metabolically active low-abundance microorganisms since the rRNA copy number in general correlates with the activity of the cell. OTU analysis also revealed that a total of 2616 OTUs were exclusively present in the rRNA datasets and not in the rDNA datasets, although there were more rDNA datasets (14) than rRNA datasets (12). It appeared that the abundance of these OTUs was too low to be detected in rDNA, but due to their high activity, they could be readily detected in rRNA.

**Table 1:** OTUs retrieved from rDNA and rRNA datasets.

**Taxon**	**rDNA & rRNA**		**rDNA**		**rRNA**	
	**Sequences (%)**	**OTUs**	**Sequences (%)**	**OTUs**	**Sequences (%)**	**OTUs**
**Archaea**	97 110 (4.91%)	170	80 069 (7.36%)	150	17 041 (1.92%)	63
**Bacteria**	1 878 896 (95.05%)	9304	1 006 483 (92.58%)	6711	872 413 (98.07%)	4020
**Unknown**	745 (0.04%)	12	604 (0.06%)	9	141 (0.01%)	4
**Total**	1 976 751	9486	1 087 156	6870	889 595	4087

**Figure 2:**
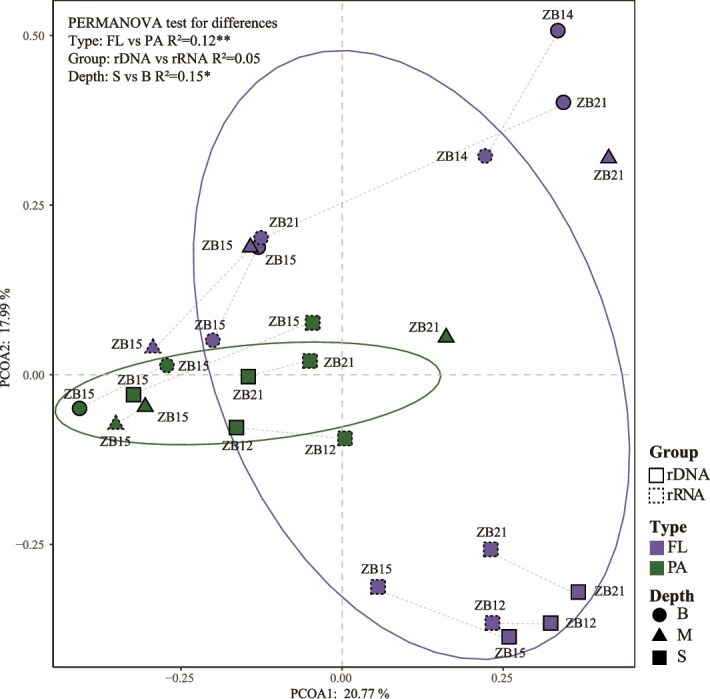
PCoA of prokaryotic communities at the OTU level based on the Bray–Curtis distance matrix. Similarity values among the samples of different biomes (“biome,” PA, and FL), water depth (“depth,” surface, middle, and bottom layer), and sample types (“type,” rDNA and rRNA) were examined using PERMANOVA test. ^*^ and ^*^^*^ indicate the significance at .05 and .01 levels, respectively.

Based on the Bray–Curtis distance matrix, the PCoA of a prokaryotic community of all samples at the OTU level showed that β-diversity values derived mostly from PA samples clustered together, indicating a high similarity in the microbial community structure among these PA samples whether rDNA or rRNA datasets were used (Fig. 2). And the dots derived from the rDNA datasets were frequently separated from those from their corresponding rRNA datasets, and in some samples, such as ZB21-BFL, they were even far apart. We speculate that these differences result from the presence of low-abundance microorganisms with high metabolic activity in the habitat.

**Figure 3:**
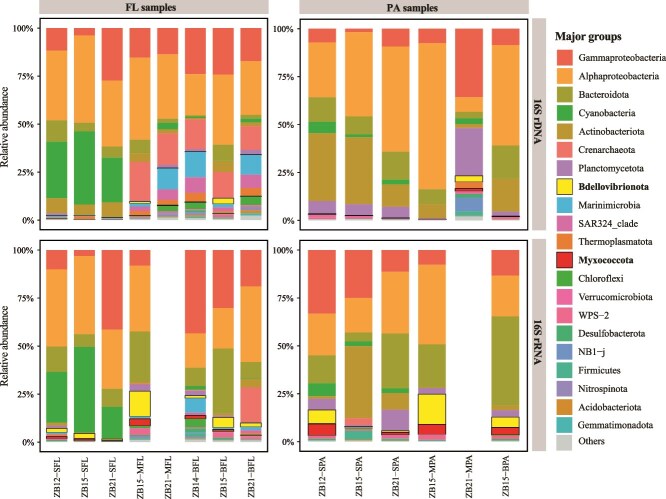
The community composition of the samples at the phylum level. The composition of 16S rDNA and rRNA samples was indicated by “DNA” and “RNA,” respectively.

**Figure 4:**
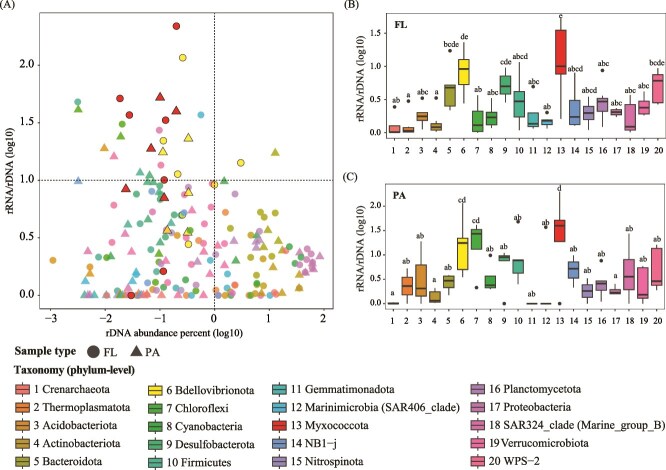
Grouping of phyla based on the abundance and activity. (A) The abundance and activity of major phyla. X-axis shows the relative rDNA abundance of major phyla in each sample by percentage. Y-axis indicates the ratios of rRNA abundance over rDNA abundance for major phyla in each sample. Horizontal dashed line indicates that the rRNA abundance equals the rDNA abundance. Vertical dashed line indicates the relative abundance of 1%. (B and C) The ratios of rRNA abundance over rDNA abundance for each major phyla in FL (B) and PA (C) samples. Values are normalized using the log10(*x* + 1) method.

More than 20 phyla were detected in both rDNA and rRNA datasets, among which phyla *Proteobacteria* (~53.6%, 50.4%), *Actinobacteriota* (~10.3%, 5.0%), *Cyanobacteria* (~7.9%, 8.4%), *Bacteroidota* (~7.6%, 18.6%), *Thaumarchaeota* (~5.6%, 1.9%), *Planctomycetota* (~3.8%, 2.8%) were predominant with both rDNA and rRNA abundances exceeding 1% (Fig. 3 and Supplementary Fig. S3). Significantly, *Bdellovibrionota* and *Myxococcota* were among the most abundance in rRNA sequences data, with their abundance >1% in over six samples (Fig. 3 and Supplementary Fig. S3; Supplementary Table S6), indicating that these phyla were metabolically active in the habitat. To correlate the abundance and activity of different phyla, we define a phylum with a relative rDNA abundance of ≥1% as a high-abundance phylum, and a phylum with a relative rRNA abundance at least 10-folds as high as the corresponding relative rDNA abundance (rRNA/rDNA ≥10) as a highly active phylum. For each phylum in each sample, the logarithmic value of its rRNA/rDNA abundance ratio plus one (one was added to allow the inclusion of the rRNA/rDNA ratio of 0 in the data analysis) was plotted against its rDNA abundance. All phyla fall into the four types, which were characterized by high abundance and high activity (X > 0, Y ≥ 1), low abundance and high activity (X < 0, Y ≥ 1), low abundance and low activity (X < 0, Y < 1), and high abundance and low activity (X > 0, Y < 1), respectively. A total of 203 data points, referred to as Phylum-Sample values (PSVs), of these four types were obtained from the 20 phyla identified in the 12 samples (Fig. 4A; Supplementary Table S7). Three out of the 203 PSVs (~1%), showing both high abundance and high activity (X > 0, Y ≥ 1), belonged to phyla *Bacteroidota*, *Cyanobacteria,* and *Bdellovibrionota*. Thirty PSVs (~15%) with low abundance and high activity (X < 0, Y ≥ 1) were mainly of phyla *Myxococcota* (8), *Bdellovibrionota* (6), and *Desulfobacterota* (4). Most PSVs were of low activity with either low abundance (X < 0, Y < 1; 101 PSVs, ~50%) or high abundance (X > 0, Y < 1; 69 PSVs, ~34%). Clearly, *Proteobacteria* and *Actinobacteriota* (in all 12 samples), as well as *Bacteroidota* (11/12), *Cyanobacteria* (7/11), and *Planctomycetota* (7/12) were abundant but relatively inactive (X > 0, Y < 1) in most of the Zhenbei seamount samples. For instance, *Proteobacteria* and *Actinobacteriota*, the most common phyla in marine habitats, exhibited an average rDNA abundance of ≥10% (X = 1.72 and 1.00, respectively), but their rRNA/rDNA ratios were <1 (Y < 0.30) (Fig. 4A; Supplementary Table S6). Notably, *Bdellovibrionota* and *Myxococcota*, the two well-known predatory bacteria in both FL and PA, and *Chloroflexi* in PA, showed remarkably higher rRNA/rDNA ratios than the other microbial phyla (Fig. 4B and C). Specifically, *Bdellovibrionota* and *Myxococcota* showed a relative rDNA abundance of <1% or even <0.1%, and their rRNA was 10- or even 200-fold more than rDNA in both FL and PA (Supplementary Table S6). These observations suggest that the predatory bacteria are highly active in the Zhenbei seamount.

### Phylogenetic diversity of the active predatory bacteria

**Figure 5:**
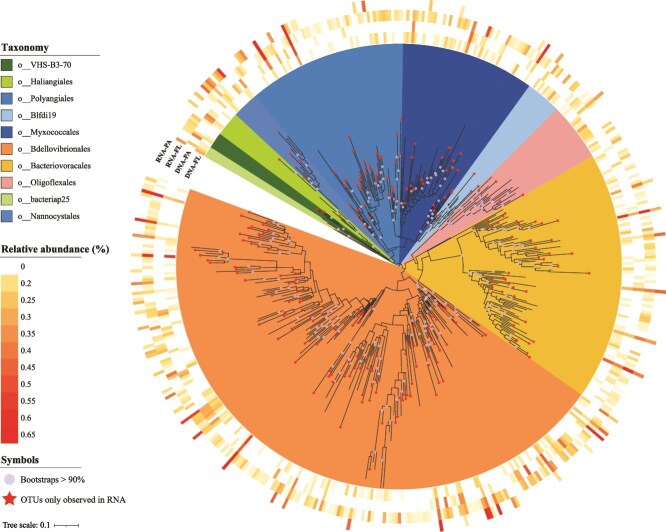
The phylogenetic tree of the OTUs of *Bdellovibrionota* and *Myxococcota.* The different orders in these phyla are indicated. The heatmap indicates the average abundance fraction of each OTUs in samples collected from different biomes (PA and FL) and nucleic acid datasets (DNA and RNA). OTUs observed only in RNA samples are labeled with red stars.

We then looked into the phylogenetic diversity of these predatory bacteria. A total of 359 *Bdellovibrionota* OTUs and 144 *Myxococcota* OTUs were detected in all 14 rDNA and 11 rRNA libraries (Table 2). Bacteria belonging to orders *Bdellovibrionales*, *Bacteriovoracia*, *Oligoflexales* in phylum *Bdellovibrionota* and orders *Polyangiales*, *Myxococcales*, *Haliangiales*, *Nannocystales*, Polyangia-Blfdi19, bacteriap25 in phylum *Myxococcota* were abundant with an average relative sequence abundance of >1% in corresponding phylum *Bdellovibrionota* and *Myxococcota* rDNA or rRNA, respectively (Fig. 5). Among them, taxa with significantly more rRNA sequences than their encoding rDNA sequences were *Bdellovibrionales* (4.03% of the total RNAs vs. 0.47% of the total rDNAs), *Bacteriovoracia* (0.42% vs. 0.19%), *Polyangiales* (1.50% vs. 0.07%), and *Myxococcales* (0.30% vs. 0.08%) (Table 2). A total of 10 OTUs (i.e. OTU27, 28, 30, 93, 101, 105, 154, 165, 168, 204) with an average rRNA abundance of >0.1% (i.e. >900 of the total 889 595 rRNA sequences) were detected in at least 6 samples (Supplementary Tables S4 and S8). They belonged to clade OM27 of the family *Bdellovibrionaceae* (OTU30, 28, 105, 165, and 204), family *Halobacteriovoraceae* (OTU101 and OTU154), families *Sandaracinaceae* (OTU27 and OTU93), and family *Myxococcaceae* (OTU168) (Supplementary Table S8). All these OTUs were remotely related to the known genera and species (85.33%–94.12% sequence identity), suggesting that predominant and active *Myxococcota* and *Bdellovibrionota* lineages in Zhenbei seamount have yet to be cultured.

**Table 2:** OTUs retrieved from the rDNA and rRNA datasets of predatory bacteria.

**Taxon**	**rDNA & rRNA**		**rDNA**		**rRNA**		**Only in rRNA**	
	**Sequences (%)**	**OTUs**	**Sequences (%)**	**OTUs**	**Sequences (%)**	**OTUs**	**Sequences**	OTUs (s)^*^
***Bdellovibrionota***	**47 243 (2.39%)**	**359**	**7455 (0.68%)**	**191**	**39 788 (4.47%)**	**250**	**2122**	**168 (12)**
***Bacteriovoracales***	5802 (0.29%)	91	2043 (0.19%)	52	3759 (0.42%)	56	176	39 (7)
***Bdellovibrionales***	41 008 (2.07%)	240	5148 (0.47%)	117	35 860 (4.03%)	183	1871	123 (12)
***Oligoflexales***	387 (0.02%)	22	245 (0.02%)	19	142 (0.02%)	8	48	3 (2)
**Other**	46 (0.002%)	6	19 (0.002%)	3	27 (0.003%)	3	27	3 (3)
***Myxococcota***	**19 855 (1.00)**	**144**	**2370 (0.22%)**	**83**	**17 485 (1.97%)**	**102**	**717**	**68 (11)**
***Myxococcales***	3538 (0.18%)	48	837 (0.08%)	27	2701 (0.30%)	34	136	21 (8)
***Haliangiales***	309 (0.02%)	7	267 (0.02%)	6	42 (0.005%)	2	25	1 (1)
***Nannocystales***	940 (0.05%)	10	170 (0.02%)	14	770 (0.09%)	7	96	3 (1)
***Polyangiales***	14 196 (0.72)	52	812 (0.07%)	19	13 384 (1.50%)	41	379	33 (11)
**VHS-B3-70**	76 (0.004%)	5	21 (0.002%)	2	55 (0.006%)	5	40	3 (2)
**Polyangia-Blfdi19**	561 (0.03%)	14	135 (0.01%)	10	426 (0.05%)	9	32	4 (3)
**bacteriap25**	235 (0.01)	8	128 (0.01%)	5	107 (0.01%)	4	9	3 (3)

Bold is the total data for phyla.

^*^Stands for the number of samples in which these OTUs have been detected.

Furthermore, 168 *Bdellovibrionota* OTUs (46.8%) and 68 *Myxococcota* OTUs (47.2%) were observed only in rRNA datasets and not in rDNA datasets, and they were mainly affiliated with *Bdellovibrionales* (123 OTUs), *Bacteriovoracia* (39 OTUs), *Polyangiales* (33 OTUs), and *Myxococcales* (21 OTUs) (Fig. 5; Table 2 and Supplementary Table S4). It is worth noting that the vast majority of these OTUs (151 *Bdellovibrionota* OTUs and 63 *Myxococcota* OTUs) were detected only in a single sample, suggesting that their metabolic activity was environmentally restricted. Only a few OTUs were found in ≥2 samples, with OTU1998, OTU369, and OTU421, all of which belonged to *Bdellovibrionota*, being the most widely distributed (in five samples) (Supplementary Tables S4 and S8). Like OTU30, OTU369, and OTU421 were affiliated with the uncultured OM27 clade of *Bdellovibrionaceae*, whereas OTU1998 might be taxonomically assigned to *Pseudobdellovibrionaceae*. Therefore, while most of these OTUs show habitat specificity, a few of them, though present at low abundance and hardly detectable at the rDNA level, represent widely distributed and metabolically active predatory bacteria in the seamount waters.

### MAGs of the predatory bacteria

**Figure 6:**
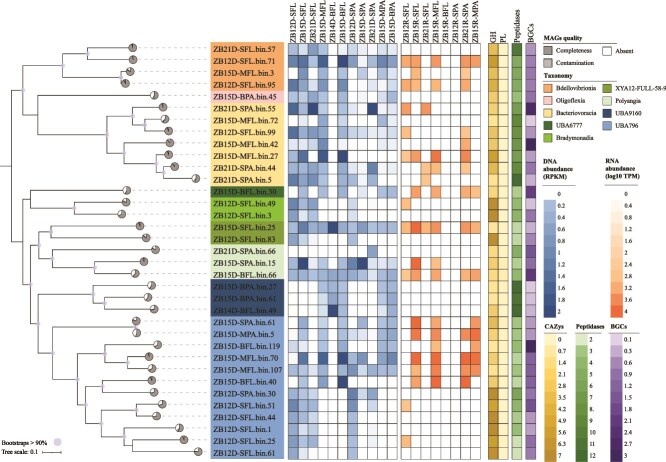
The abundance distribution of the MAGs of *Bdellovibrionota* and *Myxococcota*. The phylogenetic tree of 35 *Bdellovibrionota* and *Myxococcota* MAGs are reconstructed. Pie charts indicate the quality of each MAG. The relative abundance of each MAG in all metagenomic (DNA) and metatranscriptomic (RNA) samples, as well as the numbers of CAZy, peptidases, and BCGs related genes of each MAG are visualized in the heat maps.

We succeeded in obtaining and sequencing metagenomes from 11 samples, including both FL and PA samples from the three depths at ZB15 and the surface water at ZB12 and ZB21, and a BFL sample at ZB14. Metatranscriptomic sequencing data were obtained from eight of these samples (i.e. ZB12-SFL, SPA, ZB15-SFL, MFL, BFL, MPA, ZB21-SFL, and SPA) (Supplementary Table S9). Approximately 4 097 280 contigs with a length of ≥1 kb from metagenomes were assembled (Supplementary Table S10). A total of 444 MAGs with >50% completeness and <10% contamination was obtained through binning. Among them, 23 and 12 MAGs were assigned to the predatory bacteria *Myxococcota* and *Bdellovibrionota*, respectively (Fig. 6; Supplementary Table S11). The metabolic activity of the MAGs was then assessed by mapping with reads from metatranscriptomes. The 23 *Myxococcota* MAGs belonged to classes *Bradymonadia* (2) and *Polyangia* (3) as well as uncultured taxa XYA-12 (2), UBA6777 (1), UBA796 (12), and UBA9160 (3) (UBA: Uncultivated Bacteria and Archaea). Ten MAGs, including all three UBA9160 MAGs, four UBA796 MAGs, and one each of *Bradymonadia*, *Polyangia* and XYA-12 MAGs, were not significantly transcribed as revealed by metatranscriptomics, and their metagenomic RPKM values are lower than 0.5 in most samples, indicating the low abundance and low metabolic activity of the bacteria with these MAGs in the habitats (Fig. 6; Supplementary Table S12). The metatranscriptomic TPM and the metagenomic RPKM values of the remaining 13 MAGs varied among different samples (Fig. 6). Four MAGs, i.e. ZB15-BFL-bin30 (UBA6777), ZB15-MFL-bin107, ZB15-MFL-bin70, and ZB15-MPA-bin5 (UBA796), displayed similar expression profiles with relatively high TPMs (≥1000) in all four sequenced ZB15 samples (i.e. SFL, MFL, BFL, and MPA) (Fig. 6; Supplementary Table S12). Among them, ZB15-MFL-bin70 had the highest transcriptional abundance, with TPM values of 14 563.05, 16 001.36, 25 041.49, and 8425.39, respectively. The same observations were also made on ZB15-BFL-bin40 (TPM: 2046.29, 13 603.38, 64 560.44 in ZB15-SFL, MFL, BFL) and ZB15-SPA-bin61 (TPM: 12 776.58, 2722.126, 1817.72 in ZB15-SFL, BFL, MPA), except that the two MAGs were missing from the MPA and MFL samples, respectively. ZB15-SFL-bin25, the only MAG belonging to *Bradymonadaceae*, was highly transcribed (TPM: 12 220.93) in ZB15-SFL, and its transcription was also detected in five other samples (i.e. ZB12-SFL, ZB21-SFL, ZB15-MFL, ZB15-BFL, and ZB15-MPA). ZB15-SPA-bin15 (*Polyangiales*) was even more highly transcribed (TPM: 20 536.6) than ZB15-SFL-bin25 in ZB15-SFL. This MAG was also actively transcribed in ZB15-BFL (TPM: 863.19) but not detectably expressed in other samples. ZB15-BFL-bin66 (*Polyangiales*) was transcribed in all four sequenced ZB15 (TPM: 2359.29, 1321.38, 1824.04, and 2037.70 in SFL, MFL, BFL, and MPA, respectively) as well as ZB12-SFL (TPM: 434.32) samples (Fig. 6; Supplementary Table S12).

The 12 *Bdellovibrionota* MAGs were taxonomically assigned to three phylogenetic clades, namely class *Bacteriovoracia* (7), *Bdellovibrionia* (4), and *Oligoflexia* (1) (Supplementary Table S12). Three of the MAGs, i.e. ZB15-BPA-bin45 (*Oligoflexia*), ZB15-MFL-bin72 (*Bacteriovoracia*), and ZB21-SFL-bin57 (*Bdellovibrionia*), apparently existed in low abundance, as indicated by their low RPKM values, and few transcription products of these MAGs were detected in metatranscriptomic data (Fig. 6; Supplementary Table S12). Like *Myxococcota* ZB15-BFL-bin66, *Bdellovibrionota* MAG ZB21-SFL-bin71 was significantly transcribed in five metatranscriptomic samples, including all four sequenced ZB15 samples (TPM: 2199.52, 2653.64, 2672.13, and 2393.32 in SFL, MFL, BFL, and MPA, respectively) and a ZB12-SFL sample (TPM: 1750.91). Similar transcription profiles were also found for *Bacteriovoracia* MAG ZB15-MFL-bin27 (TPM: 2489.91, 4242.14, 6953.45, and 418.82 in ZB15-SFL, MFL, BFL, and ZB12-SFL, respectively) and *Bdellovibrionia* MAG ZB12-SFL-bin95 (TPM: 1091.04, 1671.64, 1189.27, and 1661.88 in ZB15-SFL, MFL, BFL, and ZB12R-SFL, respectively) in the above samples except for ZB15 MPA (Fig. 6). The transcripts of *Bacteriovoracia* MAG ZB21-SPA-bin44 were detected in 3 samples, i.e. ZB21-SFL (TPM: 591.31), ZB15-MFL (TPM: 485.38), and ZB15-BFL (TPM: 1062.93). We infer from these results that the abundance and activity of these predatory bacteria depend on their geographic location and lifestyle (i.e. in either a FL or a PA state).

### Gene expression and metabolic potentials of the predatory bacteria

To learn more about the metabolic potentials and activities of the predatory bacteria in the seawater around the Zhenbei seamount, the 35 MAGs of *Myxococcota* and *Bdellovibrionota* were subjected to analysis for encoded functions. As indicated by MW-scores (metabolic weight scores), these predatory bacteria were heterotrophs capable of utilizing amino acids, fatty acids, and complex carbon compounds. Unexpectedly, genes for arsenate reduction (*ars*) and sulfur oxidation (*sdo*) not only exist in 31 and 28 of the 35 MAGs, respectively (Supplementary Fig. S4) but also account for significant functional fractions, with MW-score contribution ranging from 9.9 to 10.8 and 9.8 to 10.4, respectively (Supplementary Fig. S5). It is unclear why the two pathways are active in the Zhenbei seamount.

**Figure 7:**
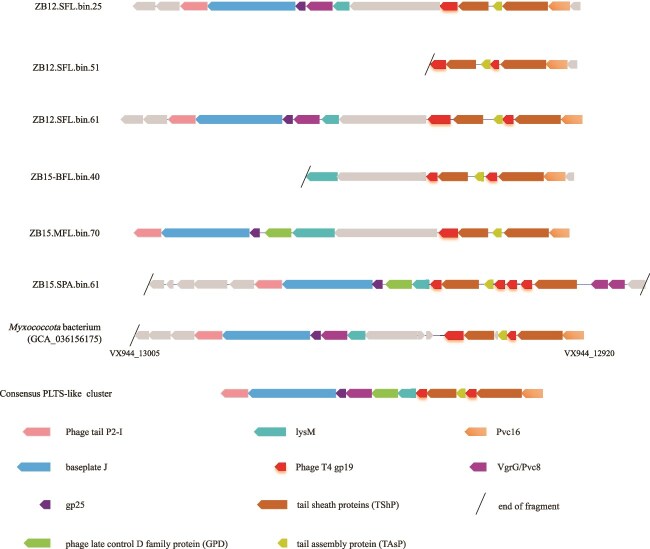
PLTS-like clusters in the *Myxococcota* MAGs. The MAGs obtained in this study and a *Myxococcota* MAG (DS_MAG26) with sequence (36 771 bp) submitted to GenBank (GCA_036156175) by Xu S. and Hou S, which has nearly 100% sequence similarity to the cluster in ZB12-SFL-bin51, are shown, together with the consensus PLTS-like cluster reported by Sarris *et al.* [68]. Genes conserved in the PLTS-like cluster are marked in colors, and hypothetical or nonconserved genes are shown in gray. The locations of the clusters are indicated on the left side. Annotation of the genes is indicated by color blocks at the bottom.

As shown above, 4 *Bdellovibrionota* MAGs and 8 *Myxococcota* MAGs exhibited highly active transcription (Supplementary Table S12). By identifying 10 most highly expressed genes from each of the MAGs, we obtained a total of 73 genes. These genes were mainly involved in transcription (9), translation (24), energy conservation (7), and nucleotide, lipid, or inorganic ion transport and metabolism (7) (Supplementary Table S13). This finding agrees with the notion that predatory bacteria are metabolically active in the habitat. Notably, genes encoding bacterial flagellum and pilus proteins, such as PilA (a type IV pilus assembly protein or a pilus assembly protein), Flp/Fap pilin component and flagellins, etc., were frequently found (Supplementary Table S14), indicating that the predators represented by these MAGs were hunting around actively, as these genes were shown to be upregulated during bacterial predation [63–67]. Intriguingly, the gene with the highest expression abundance in two *Myxococcota* MAGs (ZB15-MFL.bin.107, 5.27 Mb and ZB15-SPA.bin.61, 3.68 Mb) encoded the tail tube protein gp19 of a T4-like virus with domain Phage_T4_gp19 (Pfam PF06841) (Supplementary Table S14). This gene was also actively transcribed in seven other *Myxococcota* MAGs (Supplementary Table S15), indicating that its active transcription was not a coincidence. Further analysis revealed that the gene encoding the phage tail tube protein is often linked to a gene encoding the phage tail sheath family protein, and the two genes, along with their upstream and downstream ORFs, form a consensus PLTSs (phage-like-protein-translocation structures)-like cluster, also known as the extracellular contractile injection system (eCIS) (Fig. 7; Supplementary Table S16). Most of the other phage genes were absent, ruling out the presence of a prophage. Indeed, the gene cluster of these *Myxococcota* MAGs shares a modular organization with the antifeeding prophage (Afp) from *Serratia entomophila* and the virulence cluster from *Phogorhabdus Iuminescens* (PVC), two typical eCISs [69–71]. The eCIS homologs encoded by the MAGs were highly similar, or even identical, at the amino acid sequence level to those encoded by the *Myxococcota* MAGs previously obtained from environments, but shared low similarity with those encoded by cultured *Myxococcota* strains (Supplementary Table S16). Structural prediction with AlphaFold showed that the gp19 proteins encoded by the MAGs were most similar to the corresponding proteins of AlgoCIS, the eCIS of the marine bacterium *Algoriphagus machipongonensis* (PDB:7aeb, pTM = 0.82) [72], or PVCs from *Photorhabdus asymbiotica* (PDB:6j0b, pTM = 0.84) [73] (Supplementary Fig. S6). eCIS is believed to function as a toxin-delivery system widely used by bacteria and archaea in environments to mediate antagonistic interactions with eukaryotes and other prokaryotes [74]. Our observation suggests that this extracellular contractile injection system may play a part in predation by *Myxococcota* at seamounts.

Since predation involves the degradation of the cell wall of prey bacteria, we examined genes encoding glycoside hydrolases in the MAGs. As many as 668 genes encoding putative glycoside hydrolases (GHs) (503 GHs from *Myxococcota* and 165 GHs from *Bdellovibrionota*) of 51 GH families and 48 genes encoding polysaccharide lyases (PLs) (46 PLs from *Myxococcota* and 2 PLs from *Bdellovibrionota*) of 9 PL families were found (Supplementary Table S17). GH23 was present in all 35 MAGs with one or more copies in each MAG (102 from 23 *Myxococcota* MAGs and 71 from 12 *Bdellovibrionota* MAGs). This protein may be involved in the degradation of bacterial cell walls as a lysozyme (EC 3.2.1.17) or peptidoglycan lytic transglycosylase/peptidoglycan lyase (EC 4.2.2.29). In addition, GH102, GH103, and GH171, members of three new GH families, including cell wall degradation enzymes, were detected in 9 *Bdellovibrionota* and 18 *Myxococcota* MAGs. In most of the significantly transcribed MAGs, the expression of at least one of these genes was detected (Supplementary Table S18). The diversity and activity of these GH family proteins conceivably serve an important role in facilitating predation by the predatory bacteria.

## Discussion

16S rDNA-based analysis showed that *Proteobacteria*, *Actinobacteriota*, *Cyanobacteria*, *Bacteroidota*, *Thaumarchaeota*, and *Planctomycetota* were the dominant phyla at the Zhenbei seamount, as expected from the previous studies of seamount microbial communities [3, 15, 19, 20]. However, interesting differences existed between the rDNA- and rRNA-based microbial communities. A total of 6870 and 4087 OTUs were obtained with the rDNA- and rRNA-based approaches, respectively. As many as 2616 rRNA-based OTUs were readily identified only with the rRNA-based approach, suggesting the presence of low-abundance and high-activity OTUs in the seamount. Furthermore, according to our definition of high and low abundance and activity, most taxa were relatively inactive in most niches, while highly active taxa were mostly low in abundance at the Zhenbei seamount (Fig. 4A).

Strikingly, *Bdellovibrionota* and *Myxococcota*, two well-known predatory bacteria, were highly active in the Zhenbei seamount. This finding remains valid regardless of the amount of biomass in a sample (Supplementary Table S19). The two predators are widely distributed in various habitats but, to the best of our knowledge, have not been shown to be predominant microbes in marine waters by using rDNA-based analysis and were not often detected even in rRNA-based surveys in some oceanic habitats [16, 25, 26, 75–77]. Both *Bdellovibrionota* and *Myxococcota*, which actively hunt and consume other bacteria as their food, were initially assigned to class *Deltaproteobacteria* and recently reclassified as new phyla according to the Genome Taxonomy Database (GTDB) [78, 79]. In 2021, Petters *et al.* screened the SSU (16S and 18S) rRNA fractions of 28 soil metatranscriptome datasets from 11 different soils across Europe and found that *Myxobacteria* (currently named *Myxococcales* in phylum *Myxococcota*) accounted for 1.5%–9.7% of all SSU rRNA transcripts and >60% of all identified potential bacterivores in most soils. They suggested that *Myxobacteria* were keystone taxa in soil [80]. It has been shown that salinity is a key factor influencing the global distribution and differentiation of *Myxococcota* [81–84]. Zou *et al.* reported that the highest relative abundance of *Myxococcota* in saline habitats occurred in mangroves (1.56%), followed by coastal/estuarine (1.28%), marine (0.90%), and salt lakes (0.69%) [81]. In this study, we found that the *Myxococcota* abundance (0.02%–0.32%) in most of the samples was similar to that in other marine habitats (Fig. 3; Supplementary Table S6). In terms of community composition, Zhenbei seamount exhibits similarities with mangroves, with *Myxococcales*, *Polyangiales*, and *Haliangiales* as the predominant orders, although they differ significantly in proportion (accounting for 36.4%, 31.8%, and 9.1% of the total *Myxococcota* in Zhenbei seamount, and 16.3%, 38.1%, and 11.1% in mangroves). Additionally, *Nannocystales* (9.1%), Blfdi19 (4.5%), and Bacteriap25 (4.5%), a clade commonly distributed in terrestrial soils and freshwater sediments, were also identified in our study (Table 2), suggesting a high diversity of *Myxococcota* in Zhenbei seamount. *Bdellovibrionota*, also known as *Bdellovibrio* and like organisms, currently comprises *Bdellovibrionia*, *Bacteriovoracales*, *Oligoflexia*, and other members that lack a taxonomic assignment and can be found in various environments [75, 85–88]. Based on the analysis of 16S miTags from the Tara Ocean and Mariana marine water metagenomes, *Bacteriovoracia* and Bdello-group2, which consists of representative *Bdellovibrio* predators within *Bdellovibrionales*, were relatively abundant in oceans, while *Oligoflexia* and Bdello-group1 were very low in abundance in the marine environment [75]. Similarly, the relative abundance of *Oligoflexia* (2.9% of the *Bdellovibrionota*) was significantly lower than that of *Bdellovibrionales* (69.1%) and *Bacteriovoracales* (11.3%) in Zhenbei seamount (Table 2). Because of the lack of extensive rRNA diversity studies, it remains to be understood if active microbial predating observed at Zhenbei seamount occurs in other marine habitats.

Our findings suggest that the seamounts are hunting grounds for microbial predators. As a unique ecosystem where POM and inorganic nutrients are enriched, a seamount is a hot spot for marine microflora. Microorganisms thriving at seamounts are potential prey for predatory bacteria. It is worth noting, however, that the activity of predatory bacteria did not appear to correlate with the measured geochemical parameters, as revealed in the study (Supplementary Tables S1 and S6). The possibility exists that the hunting activity of the microbial predators depends on the amounts of their prey rather than the total biomass. It is known that the strength and persistence of the hydrological effects of seamounts exhibit substantial spatiotemporal variation [89]. During July, when the samples for this study were collected, typical meteorological and hydrological conditions in the SCS, including high temperature, prevalent monsoon/typhoons, and other types of volatile climate, would intensify the seamount effects [90], thereby enhancing microbial predatory activities. Therefore, while active hunting by microbial predators facilitated by the seamount effects would conceivably occur in all seasons, the intensity of the hunting may vary with spatiotemporal changes.

*Bdellovibrionota* and *Myxococcota* represent two bacterial phyla with distinct predation strategies [76–78, 86]. Indeed, we found that the MAGs of the two predatory bacteria encode a range of carbohydrate-hydrolyzing enzymes, with putative cell wall-degrading glycoside hydrolases of the GH23, GH102, and GH171 families being the most abundant (Supplementary Table S17) [77, 91]. It has been shown that genes encoding flagella and pili are expressed during the early interaction of the predatory bacteria with the prey [63–67]. In this study, transcriptional analysis of the 35 MAGs of *Bdellovibrionota* and *Myxococcota* shows that genes encoding flagellum and pilus proteins were, in general, highly expressed (Supplementary Table S14), suggesting that these predators were actively hunting their prey through increased motility.

Strikingly, a gene encoding the T4-like virus tail tube protein gp19 is encoded and highly expressed in several *Myxococcota* MAGs, most of which belong to uncultivated UBA796 (Supplementary Table S15). Judging from sequence similarity, structural prediction, and genome neighbors of its encoding gene, we suggest that this tube protein is an eCIS protein. Chen *et al.* identified 631 eCIS-like loci, including those from three *Myxococcota* strains (*Corallococcus coralloides* DSM 2259, *Myxococcus stipitatus* DSM 14675, *Myxococcus fulvus* 124B2), from 11 699 publicly available complete bacterial and archaea genomes [92]. Despite the widespread occurrence, this study provides the first report of the high expression of an eCIS gene from an environment sample. The eCIS is assembled in the bacterial cytoplasm of the donor cell and subsequently released into the extracellular space, where it binds to the surface of a target cell, contracts and punctures the cell envelope, and injects the effector proteins [69, 72, 73, 93]. Therefore, it is tempting to speculate that the eCIS is one of the weapons employed by the bacterial predators in attacking the prey during predation. *Myxococcota* bacteria, which have yet to be cultured, are likely active in hunting in seamounts.

Bacterial predators play an important role in structuring microbial communities and maintaining biodiversity in various environments [76, 80]. Hungate *et al.* found that predators grow, metabolize, and feed at a higher rate than most bacteria in the soil food web, exerting a top-down effect in microbial food chains [76, 94, 95]. Chen *et al.* showed that *Halobacteriovorax*, a genus of the predatory bacteria within *Bdellovibrionota* and ubiquitous in saltwater environments, was most responsive to the prey *Vibrio vulnificus*, increasing by 18-fold with a concomitant reduction of its prey by 4–5 orders of magnitude in a seawater microcosm [96]. It appears that predatory bacteria serve a similar role in the seamount microbiome. The hunting activity of predatory bacteria in seamount environments, mediated by cell wall hydrolase and the eCIS, accelerates the death of their prey and the changes in the microbial communities. Unlike what is observed in higher organisms, slow-growing bacteria are more likely to survive predation by bacterial predators than fast-growing bacteria [97, 98]. Thus, predators may limit the dominance of species with *r*-strategy, which grow rapidly and dominate in the community when resources are temporarily abundant [98]. This provides an explanation for the low activity of most phyla, despite their rDNA abundance, in the presence of active predatory bacteria as observed in this study (Fig. 3). We infer from our results that seamounts may serve as hunting grounds for predatory bacteria, and bacterial predation will impact the element and energy flows in shaping the seamount ecosystems.

## Supplementary Material

Supplementary_Materials_ycaf042

Supplementary-tables_ycaf042

## Data Availability

The HiSeq sequencing data for 16S rRNA gene libraries, raw reads of metagenomic and metatranscriptomic data obtained in this study were deposited in the National Omics Data Encyclopedia (NODE) database with the BioProject accession number OEP00005639 (https://www.biosino.org/node/project/detail/OEP00005639).

## References

[1] Yesson C, Clark MR, Taylor ML et al. The global distribution of seamounts based on 30 arc seconds bathymetry data. *Deep-Sea Res I Oceanogr Res Pap* 2011;58:442–53. 10.1016/j.dsr.2011.02.004

[2] Staudigel H, Koppers AAP, Lavelle JW et al. Defining the word “Seamount”. *Oceanography* 2010;23:20, 20–1. 10.5670/oceanog.2010.85

[3] Li H, Zhou H, Yang S et al. Stochastic and deterministic assembly processes in seamount microbial communities. *Appl Environ Microbiol* 2023;89:e0070123. 10.1128/aem.00701-23 37404136 PMC10370332

[4] Rogers AD . The biology of seamounts: 25 years on. Sheppard C (ed.), In: Advances in Marine Biology, Vol. 79. Amsterdam, The Netherlands: Elsevier, 2018, 137–224. ISBN 9780128151013. 30012275 10.1016/bs.amb.2018.06.001

[5] Baletaud F, Lecellier G, Gilbert A et al. Comparing seamounts and coral reefs with eDNA and BRUVS reveals oases and refuges on shallow seamounts. *Biology* 2023;12:1446. 10.3390/biology12111446 37998045 PMC10669620

[6] Zhao R, Zhao F, Zheng S et al. Bacteria, protists, and fungi may hold clues of seamount impact on diversity and connectivity of deep-sea pelagic communities. *Front Microbiol* 2022;13:773487. 10.3389/fmicb.2022.773487 35464911 PMC9024416

[7] Letessier TB, Mouillot D, Bouchet PJ et al. Remote reefs and seamounts are the last refuges for marine predators across the Indo-Pacific. *PLoS Biol* 2019;17:e3000366. 10.1371/journal.pbio.3000366 31386657 PMC6684043

[8] Nishida K, Murakami C, Yonezaki S et al. Prey use by three deep-sea fishes in the Emperor Seamount waters, North Pacific Ocean, as revealed by stomach contents and stable isotope analyses. *Environ Biol Fish* 2016;99:335–49. 10.1007/s10641-016-0477-x

[9] Mendonça A, Arístegui J, Vilas JC et al. Is there a seamount effect on microbial community structure and biomass? The case study of Seine and Sedlo Seamounts (Northeast Atlantic). *PLoS One* 2012;7:e29526. 10.1371/journal.pone.0029526 22279538 PMC3261146

[10] Rowden AA, Schlacher TA, Williams A et al. A test of the seamount oasis hypothesis: seamounts support higher epibenthic megafaunal biomass than adjacent slopes. *Mar Ecol* 2010;31:95–106. 10.1111/j.1439-0485.2010.00369.x

[11] Morato T, Hoyle SD, Allain V et al. Seamounts are hotspots of pelagic biodiversity in the open ocean. *Proc Natl Acad Sci* 2010;107:9707–11. 10.1073/pnas.0910290107 20448197 PMC2906904

[12] Stocks KI, Clark MR, Rowden AA et al. CenSeam, an international program on seamounts within the census of marine life: achievements and lessons learned. *PLoS One* 2012;7:e32031. 10.1371/journal.pone.0032031 22312448 PMC3270038

[13] Clark MR, Rowden AA, Schlacher T et al. The ecology of seamounts: structure, function, and human impacts. *Annu Rev Mar Sci* 2010;2:253–78. 10.1146/annurev-marine-120308-081109 21141665

[14] Doyle SM, Self MJ, Hayes J et al. Microbial community dynamics provide evidence for hypoxia during a coral reef mortality event. *Appl Environ Microbiol* 2022;88:e0034722. 10.1128/aem.00347-22 35435720 PMC9088274

[15] Yu M, Zhang M, Zeng R et al. Diversity and potential host-interactions of viruses inhabiting deep-sea seamount sediments. *Nat Commun* 2024;15:3228. 10.1038/s41467-024-47600-1 38622147 PMC11018836

[16] Xu S, Huang H, Chen S et al. Recovery of 1887 metagenome-assembled genomes from the South China Sea. *Sci Data* 2024;11:197. 10.1038/s41597-024-03050-4 38351104 PMC10864278

[17] Sun J, Zhou H, Cheng H et al. Depth-dependent distribution of prokaryotes in sediments of the manganese crust on Nazimov Guyots of the Magellan Seamounts. *Microb Ecol* 2023;86:3027–42. 10.1007/s00248-023-02305-8 37792089

[18] Huang H, Xu S, Li S et al. Diversity and distribution of harmful algal bloom species from seamount to coastal waters in the South China Sea. *Microbiol Spectr* 2023;11:e0416922. 10.1128/spectrum.04169-22 36815795 PMC10100961

[19] Busch K, Hanz U, Mienis F et al. On giant shoulders: how a seamount affects the microbial community composition of seawater and sponges. *Biogeosciences* 2020;17:3471–86. 10.5194/bg-17-3471-2020

[20] Djurhuus A, Boersch-Supan PH, Mikalsen S-O et al. Microbe biogeography tracks water masses in a dynamic oceanic frontal system. *R Soc Open Sci* 2017;4:170033. 10.1098/rsos.170033 PMC5383857 28405400

[21] Khandeparker R, Meena RM, Deobagkar D. Bacterial diversity in deep-sea sediments from Afanasy Nikitin Seamount, Equatorial Indian Ocean. *Geomicrobiol J* 2014;31:942–9. 10.1080/01490451.2014.918214

[22] Zhang X, Wu K, Han Z et al. Microbial diversity and biogeochemical cycling potential in deep-sea sediments associated with seamount, trench, and cold seep ecosystems. *Front Microbiol* 2022;13:1029564. 10.3389/fmicb.2022.1029564 36386615 PMC9650238

[23] Liu H, Jing H, Wang F. Archaea predominate in the ammonia oxidation process in the sediments of the Yap and Mariana Trenches. *Front Microbiol* 2023;14:1268790. 10.3389/fmicb.2023.1268790 PMC10568479 37840747

[24] Luo Y, Wei X, Yang S et al. Fungal diversity in deep-sea sediments from the Magellan seamounts as revealed by a metabarcoding approach targeting the ITS2 regions. *Mycology* 2020;11:214–29. 10.1080/21501203.2020.1799878 33062383 PMC7534268

[25] Liu R, Wang Z, Wang L et al. Bulk and active sediment prokaryotic communities in the Mariana and Mussau Trenches. *Front Microbiol* 2020;11:1521. 10.3389/fmicb.2020.01521 PMC7381213 32765444

[26] Li R, Tun HM, Jahan M et al. Comparison of DNA-, PMA-, and RNA-based 16S rRNA Illumina sequencing for detection of live bacteria in water. *Sci Rep* 2017;7:5752. 10.1038/s41598-017-02516-3 28720878 PMC5515937

[27] Blazewicz SJ, Barnard RL, Daly RA et al. Evaluating rRNA as an indicator of microbial activity in environmental communities: limitations and uses. *The ISME J.* 2013;7:2061–8. 10.1038/ismej.2013.102 23823491 PMC3806256

[28] Jones SE, Lennon JT. Dormancy contributes to the maintenance of microbial diversity. *Proc Natl Acad Sci* 2010;107:5881–6. 10.1073/pnas.0912765107 20231463 PMC2851880

[29] Lankiewicz TS, Cottrell MT, Kirchman DL. Growth rates and rRNA content of four marine bacteria in pure cultures and in the Delaware estuary. *ISME J* 2016;10:823–32. 10.1038/ismej.2015.156 26394004 PMC4796920

[30] Kerkhof L, Ward BB. Comparison of nucleic acid hybridization and fluorometry for measurement of the relationship between RNA/DNA ratio and growth rate in a marine bacterium. *Appl Environ Microbiol* 1993;59:1303–9. 10.1128/aem.59.5.1303-1309.1993 16348926 PMC182081

[31] Kemp PF, Lee S, Laroche J. Estimating the growth rate of slowly growing marine bacteria from RNA content. *Appl Environ Microbiol* 1993;59:2594–601. 10.1128/aem.59.8.2594-2601.1993 16349018 PMC182325

[32] Yanagawa K, Morono Y, de Beer D et al. Metabolically active microbial communities in marine sediment under high-CO_2_ and low-pH extremes. *ISME J* 2013;7:555–67. 10.1038/ismej.2012.124 23096400 PMC3578575

[33] Hunt DE, Lin Y, Church MJ et al. Relationship between abundance and specific activity of bacterioplankton in open ocean surface waters. *Appl Environ Microbiol* 2013;79:177–84. 10.1128/aem.02155-12 23087033 PMC3536108

[34] Wang Z, Wang L, Liu R et al. Community structure and activity potentials of archaeal communities in hadal sediments of the Mariana and Mussau trenches. *Mar Life Sci Technol* 2022;4:150–61. 10.1007/s42995-021-00105-y 37073355 PMC10077302

[35] Hoffmann K, Hassenrück C, Salman-Carvalho V et al. Response of bacterial communities to different detritus compositions in Arctic deep-sea sediments. *Front Microbiol* 2017;8:266. 10.3389/fmicb.2017.00266 PMC5323390 28286496

[36] Campbell BJ, Kirchman DL. Bacterial diversity, community structure and potential growth rates along an estuarine salinity gradient. *ISME J* 2013;7:210–20. 10.1038/ismej.2012.93 22895159 PMC3526181

[37] Matheus , Carnevali PB, Herbold CW, Hand KP et al. Distinct microbial assemblage structure and archaeal diversity in sediments of Arctic Thermokarst Lakes differing in methane sources. *Front Microbiol* 2018;9:1192. 10.3389/fmicb.2018.01192 PMC6000721 29930542

[38] Steven B, Hesse C, Soghigian J et al. Simulated rRNA/DNA ratios show potential to misclassify active populations as dormant. *Appl Environ Microbiol* 2017;83:e00696–17. 10.1128/aem.00696-17 PMC5440720 28363969

[39] Campbell BJ, Yu L, Heidelberg JF et al. Activity of abundant and rare bacteria in a coastal ocean. *Proc Natl Acad Sci* 2011;108:12776–81. 10.1073/pnas.1101405108 21768380 PMC3150899

[40] Zhang Y, Zhao Z, Dai M et al. Drivers shaping the diversity and biogeography of total and active bacterial communities in the South China Sea. *Mol Ecol* 2014;23:2260–74. 10.1111/mec.12739 24684298 PMC4230472

[41] Zhao H, Zhang Z, Nair S et al. Vertically exported phytoplankton (< 20 μm) and their correlation network with bacterioplankton along a deep-sea seamount. *Front Mar Sci* 2022;9:862494. 10.3389/fmars.2022.862494

[42] Ma J, Song J, Li X et al. Control factors of DIC in the Y3 seamount waters of the Western Pacific Ocean. *J Oceanol Limnol* 2020;38:1215–24. 10.1007/s00343-020-9314-3

[43] Zuo J, Song J, Yuan H et al. Impact of Kuroshio on the dissolved oxygen in the East China Sea region. *J Oceanol Limnol* 2018;37:513–24. 10.1007/s00343-019-7389-5

[44] Ma J, Song J, Li X et al. Environmental characteristics in three seamount areas of the Tropical Western Pacific Ocean: focusing on nutrients. *Mar Pollut Bull* 2019;143:163–74. 10.1016/j.marpolbul.2019.04.045 31789152

[45] Ma J, Wen LL, Li XG et al. Different fates of particulate matters driven by marine hypoxia: a case study of oxygen minimum zone in the Western Pacific. *Mar Environ Res* 2024;200:106648. 10.1016/j.marenvres.2024.106648 39043062

[46] Bolyen E, Rideout JR, Dillon MR et al. Reproducible, interactive, scalable and extensible microbiome data science using QIIME 2. *Nat Biotechnol* 2019;37:852–7. 10.1038/s41587-019-0209-9 31341288 PMC7015180

[47] Martin M . Cutadapt removes adapter sequences from high-throughput sequencing reads. *EMBnetjournal* 2011;17:10. 10.14806/ej.17.1.200

[48] Callahan BJ, McMurdie PJ, Rosen MJ et al. DADA2: high-resolution sample inference from Illumina amplicon data. *Nat Methods* 2016;13:581–3. 10.1038/nmeth.3869 27214047 PMC4927377

[49] Letunic I, Bork P. Interactive Tree Of Life (iTOL) v5: an online tool for phylogenetic tree display and annotation. *Nucleic Acids Res* 2021;49:W293–6. 10.1093/nar/gkab301 33885785 PMC8265157

[50] Uritskiy GV, DiRuggiero J, Taylor J. MetaWRAP – a flexible pipeline for genome-resolved metagenomic data analysis. *Microbiome* 2018;6:158. 10.1186/s40168-018-0541-1 30219103 PMC6138922

[51] Li D, Liu CM, Luo R et al. MEGAHIT: an ultra-fast single-node solution for large and complex metagenomics assembly via succinct *de Bruijn* graph. *Bioinformatics* 2015;31:1674–6. 10.1093/bioinformatics/btv033 25609793

[52] Kang DD, Li F, Kirton E et al. MetaBAT 2: an adaptive binning algorithm for robust and efficient genome reconstruction from metagenome assemblies. *PeerJ* 2019;7:e7359. 10.7717/peerj.7359 31388474 PMC6662567

[53] Parks DH, Imelfort M, Skennerton CT et al. CheckM: assessing the quality of microbial genomes recovered from isolates, single cells, and metagenomes. *Genome Res* 2015;25:1043–55. 10.1101/gr.186072.114 25977477 PMC4484387

[54] Olm MR, Brown CT, Brooks B et al. dRep: a tool for fast and accurate genomic comparisons that enables improved genome recovery from metagenomes through de-replication. *ISME J.* 2017;11:2864–8. 10.1038/ismej.2017.126 28742071 PMC5702732

[55] Chaumeil PA, Mussig AJ, Hugenholtz P et al. GTDB-Tk v2: memory friendly classification with the genome taxonomy database. *Bioinformatics* 2022;38:5315–6. 10.1093/bioinformatics/btac672 36218463 PMC9710552

[56] Huerta-Cepas J, Forslund K, Coelho LP et al. Fast genome-wide functional annotation through orthology assignment by eggNOG-Mapper. *Mol Biol Evol* 2017;34:2115–22. 10.1093/molbev/msx148 28460117 PMC5850834

[57] Zhou Z, Tran PQ, Breister AM et al. METABOLIC: high-throughput profiling of microbial genomes for functional traits, metabolism, biogeochemistry, and community-scale functional networks. *Microbiome* 2022;10:33. 10.1186/s40168-021-01213-8 35172890 PMC8851854

[58] Blin K, Shaw S, Kloosterman AM et al. antiSMASH 6.0: improving cluster detection and comparison capabilities. *Nucleic Acids Res* 2021;49:W29–35. 10.1093/nar/gkab335 33978755 PMC8262755

[59] Kopylova E, Noe L, Touzet H. SortMeRNA: fast and accurate filtering of ribosomal RNAs in metatranscriptomic data. *Bioinformatics* 2012;28:3211–7. 10.1093/bioinformatics/bts611 23071270

[60] Nguyen LT, Schmidt HA, von Haeseler A et al. IQ-TREE: a fast and effective stochastic algorithm for estimating maximum-likelihood phylogenies. *Mol Biol Evol* 2015;32:268–74. 10.1093/molbev/msu300 25371430 PMC4271533

[61] Tria FDK, Landan G, Dagan T. Phylogenetic rooting using minimal ancestor deviation. *Nat Ecol Evol* 2017;1:193. 10.1038/s41559-017-0193 29388565

[62] Kalyaanamoorthy S, Minh BQ, Wong TKF et al. ModelFinder: fast model selection for accurate phylogenetic estimates. *Nat Methods* 2017;14:587–9. 10.1038/nmeth.4285 28481363 PMC5453245

[63] Contreras-Moreno FJ, Pérez J, Muñoz-Dorado J et al. *Myxococcus xanthus* predation: an updated overview. *Front Microbiol* 2024;15:1339696. 10.3389/fmicb.2024.1339696 38328431 PMC10849154

[64] Pérez J, Contreras-Moreno FJ, Muñoz-Dorado J et al. Development versus predation: transcriptomic changes during the lifecycle of *Myxococcus xanthus*. *Front Microbiol* 2022;13:1004476. 10.3389/fmicb.2022.1004476 36225384 PMC9548883

[65] Avidan O, Petrenko M, Becker R et al. Identification and characterization of differentially-regulated Type IVb pilin genes necessary for predation in obligate bacterial predators. *Sci Rep* 2017;7:1013. 10.1038/s41598-017-00951-w PMC5430801 28432347

[66] Karunker I, Rotem O, Dori-Bachash M et al. A global transcriptional switch between the attack and growth forms of *Bdellovibrio bacteriovorus*. *PLoS One* 2013;8:e61850. 10.1371/journal.pone.0061850 23613952 PMC3627812

[67] Lambert C, Chang CY, Capeness MJ et al. The first bite – profiling the predatosome in the bacterial pathogen *Bdellovibrio*. *PLoS One* 2010;5:e8599. 10.1371/journal.pone.0008599 20062540 PMC2797640

[68] Sarris PF, Ladoukakis ED, Panopoulos NJ et al. A phage tail-derived element with wide distribution among both prokaryotic domains: a comparative genomic and phylogenetic study. *Genome Biol Evol* 2014;6:1739–47. 10.1093/gbe/evu136 25015235 PMC4122934

[69] Hurst MR, Beard SS, Jackson TA et al. Isolation and characterization of the *Serratia entomophila* antifeeding prophage. *FEMS Microbiol Lett* 2007;270:42–8. 10.1111/j.1574-6968.2007.00645.x 17263838

[70] Yang G, Dowling AJ, Gerike U et al. Photorhabdus virulence cassettes confer injectable insecticidal activity against the Wax Moth. *J Bacteriol* 2006;188:2254–61. 10.1128/jb.188.6.2254-2261.2006 16513755 PMC1428146

[71] Hurst MR, Glare TR, Jackson TA. Cloning *Serratia entomophila* antifeeding genes – a putative defective prophage active against the grass grub *Costelytra zealandica*. *J Bacteriol* 2004;186:5116–28. 10.1128/jb.186.15.5116-5128.2004 15262948 PMC451664

[72] Xu J, Ericson CF, Lien Y-W et al. Identification and structure of an extracellular contractile injection system from the marine bacterium *Algoriphagus machipongonensis*. *Nat Microbiol* 2022;7:397–410. 10.1038/s41564-022-01059-2 35165385 PMC8894135

[73] Jiang F, Li N, Wang X et al. Cryo-EM structure and assembly of an extracellular contractile injection system. *Cell* 2019;177:370–83.e15. 10.1016/j.cell.2019.02.020 30905475

[74] Patz S, Becker Y, Richert-Pöggeler KR et al. Phage tail-like particles are versatile bacterial nanomachines – a mini-review. *J Adv Res* 2019;19:75–84. 10.1016/j.jare.2019.04.003 31341672 PMC6629978

[75] Li Q-M, Zhou Y-L, Wei Z-F et al. Phylogenomic insights into distribution and adaptation of *Bdellovibrionota* in marine waters. *Microorganisms* 2021;9:757. 10.3390/microorganisms9040757 PMC8067016 33916768

[76] Hungate BA, Marks JC, Power ME et al. The functional significance of bacterial predators. *MBio* 2021;12:e00466–21. 10.1128/mBio.00466-21 PMC8092244 33906922

[77] Pérez J, Moraleda-Muñoz A, Marcos-Torres FJ et al. Bacterial predation: 75 years and counting! *Environ Microbiol* 2016;18:766–79. 10.1111/1462-2920.13171 26663201

[78] Kamada S, Wakabayashi R, Naganuma T. Phylogenetic revisit to a review on predatory bacteria. *Microorganisms* 2023;11:1673. 10.3390/microorganisms11071673 PMC10385382 37512846

[79] Waite DW, Chuvochina M, Pelikan C et al. Proposal to reclassify the proteobacterial classes *Deltaproteobacteria* and *Oligoflexia*, and the phylum *Thermodesulfobacteria* into four phyla reflecting major functional capabilities. *Int J Syst Evol Microbiol* 2020;70:5972–6016. 10.1099/ijsem.0.004213 33151140

[80] Petters S, Groß V, Söllinger A et al. The soil microbial food web revisited: predatory myxobacteria as keystone taxa? *The ISME J.* 2021;15:2665–75. 10.1038/s41396-021-00958-2 33746204 PMC8397742

[81] Zou D, Zhang C, Liu Y et al. Biogeographical distribution and community assembly of *Myxococcota* in mangrove sediments. *Environ Microbiome* 2024;19:47. 10.1186/s40793-024-00593-2 39003484 PMC11245791

[82] Wang J, Wang J, Wu S et al. Global geographic diversity and distribution of the myxobacteria. *Microbiology*. *Spectrum* 2021;9:e0001221. 10.1128/Spectrum.00012-21 PMC8552515 34259548

[83] Chen H, Athar R, Zheng G et al. Prey bacteria shape the community structure of their predators. *ISME J* 2011;5:1314–22. 10.1038/ismej.2011.4 21326335 PMC3146273

[84] Jiang D-M, Kato C, Zhou X-W et al. Phylogeographic separation of marine and soil myxobacteria at high levels of classification. *ISME J.* 2010;4:1520–30. 10.1038/ismej.2010.84 20596070

[85] Wei T-S, Gao Z-M, Gong L et al. Genome-centric view of the microbiome in a new deep-sea glass sponge species Bathydorus sp. *Front Microbiol* 2023;14:1078171. 10.3389/fmicb.2023.1078171 PMC9944714 36846759

[86] Williams HN, Chen H. Environmental regulation of the distribution and ecology of *Bdellovibrio* and like organisms. *Front Microbiol* 2020;11:545070. 10.3389/fmicb.2020.545070 33193128 PMC7658600

[87] Ezzedine JA, Jacas L, Desdevises Y et al. *Bdellovibrio* and like organisms in Lake Geneva: an unseen elephant in the room? *Front Microbiol* 2020;11:98. 10.3389/fmicb.2020.00098 PMC7034301 32117128

[88] Crossman LC, Chen H, Cerdeño-Tárraga A-M et al. A small predatory core genome in the divergent marine *Bacteriovorax marinus* SJ and the terrestrial *Bdellovibrio bacteriovorus*. *ISME J* 2013;7:148–60. 10.1038/ismej.2012.90 22955231 PMC3526173

[89] White M, Bashmachnikov I, Arstegui J et al. Physical processes and seamount productivity. Seamounts: Ecology, Fisheries & Conservation Oxford, UK: Blackwell Publishing. 62–84. 10.1002/9780470691953.ch4

[90] Tan H-J, Cai R-S, Wu R-G. Summer marine heatwaves in the South China Sea: trend, variability and possible causes. *Adv Clim Change Res* 2022;13:323–32. 10.1016/j.accre.2022.04.003

[91] Zhang L, Dong C, Wang J et al. Predation of oomycetes by myxobacteria via a specialized CAZyme system arising from adaptive evolution. *The ISME J.* 2023;17:1089–103. 10.1038/s41396-023-01423-y 37156836 PMC10284895

[92] Chen L, Song N, Liu B et al. Genome-wide identification and characterization of a superfamily of bacterial extracellular contractile injection systems. *Cell Rep* 2019;29:511–21.e2. 10.1016/j.celrep.2019.08.096 31597107 PMC6899500

[93] Casu B, Sallmen JW, Schlimpert S et al. Cytoplasmic contractile injection systems mediate cell death in *Streptomyces*. *Nat Microbiol* 2023;8:711–26. 10.1038/s41564-023-01341-x 36894633 PMC10066040

[94] Cohen Y, Pasternak Z, Müller S et al. Community and single cell analyses reveal complex predatory interactions between bacteria in high diversity systems. *Nat Commun* 2021;12:12. 10.1038/s41467-021-25824-9 34531395 PMC8446003

[95] Chauhan A, Cherrier J, Williams HN. Impact of sideways and bottom-up control factors on bacterial community succession over a tidal cycle. *Proc Natl Acad Sci USA* 2009;106:4301–6. 10.1073/pnas.0809671106 19251645 PMC2657424

[96] Chen H, Laws EA, Martin JL et al. Relative contributions of *Halobacteriovorax* and bacteriophage to bacterial cell death under various environmental conditions. *mBio* 2018;9:e01202–18. 10.1128/mBio.01202-18 PMC6083911 30087166

[97] Apple JK, Strom SL, Palenik B et al. Variability in protist grazing and growth on different marine *Synechococcus* isolates. *Appl Environ Microbiol* 2011;77:3074–84. 10.1128/aem.02241-10 21398485 PMC3126417

[98] Lebaron P, Servais P, Troussellier M et al. Microbial community dynamics in Mediterranean nutrient-enriched seawater mesocosms: changes in abundances, activity and composition. *FEMS Microbiol Ecol* 2001;34:255–66. 10.1111/j.1574-6941.2001.tb00776.x 11137605

