# Supplementary material for: A hunting ground for predatory bacteria at the Zhenbei seamount in the South China Sea
Source: ISME Commun. 2025 Mar 5;5(1):ycaf042. doi: 10.1093/ismeco/ycaf042 (PMC11937823; doi:10.1093/ismeco/ycaf042)
Supplement: Supplementary_Materials_ycaf042 [file supplementary_materials_ycaf042.pdf]

Supplementary Materials for

A hunting ground for predatory bacteria at the Zhenbei seamount in the South China Sea

Zhimeng Li[1,2‡], Dayu Zou[3‡], Rulong Liu[4‡], Juntong Pan[2,8], Junkai Huang[1,2], Jun Ma[5], Liting Huang[4], Jiani He[4], Lulu Fu[6], Xiaowei Zheng[2], Minxiao Wang[6], Jiasong Fang[4], Hailiang Dong[8], Meng Li[3*], Li Huang[1,2,7*], Xin Dai[2,7*]

[1]Southern Marine Science and Engineering Guangdong Laboratory (Guangzhou), No. 1119 Haibin Road, Nansha, Guangzhou 511458, China.

[2]State Key Laboratory of Microbial Resources, Institute of Microbiology, Chinese Academy of Sciences, No. 1 West Beichen Road, Chaoyang District, Beijing 100101, China.

[3]Archaeal Biology Center, Synthetic Biology Research Center, Shenzhen Key Laboratory of Marine Microbiome Engineering, Key Laboratory of Marine Microbiome Engineering of Guangdong Higher Education Institutes, Institute for Advanced Study, Shenzhen University, 3688 Nanhai Avenue, Shenzhen 518060, China.

[4]College of Oceanography and Ecological Science, Shanghai Ocean University, No. 999 Huchenghuan Rd, Shanghai 201306, China.

[5]CAS Key Laboratory of Marine Ecology and Environmental Sciences, Institute of Oceanology, Chinese Academy of Sciences, 7 Nanhai Road, Qingdao 266071, China.

[6]Center of Deep-Sea Research, Institute of Oceanology, Chinese Academy of Sciences, 7 Nanhai Road, Qingdao 266071, China.

[7]College of Life Sciences, University of Chinese Academy of Sciences, No. 1 Yanqihu East Rd, Beijing 100049, China.

[8]Center for Geomicrobiology and Biogeochemistry Research, State Key Laboratory of Geomicrobiology and Environmental Changes, China University of Geosciences, No. 29 Xueyuan Road, Beijing 100083, China.

*Corresponding author: Xin Dai, State Key Laboratory of Microbial Resources, Institute of Microbiology, Chinese Academy of Sciences, No. 1 West Beichen Road, Chaoyang District, Beijing 100101, China. Email: daixin@im.ac.cn; Li Huang, Southern Marine Science and Engineering Guangdong Laboratory (Guangzhou), No. 1119 Haibin Road, Nansha, Guangzhou 511458, China. Email: huang_li@gmlab.ac.cn; Meng Li, Institute for Advanced Study, Shenzhen University, 3688 Nanhai Avenue, Shenzhen 518060, China. Email: limeng848@szu.edu.cn.

‡Zhimeng Li, Dayu Zou, and Rulong Liu contributed equally to this work.

Running Title: Bacterial predation at seamounts

Supplementary Figures

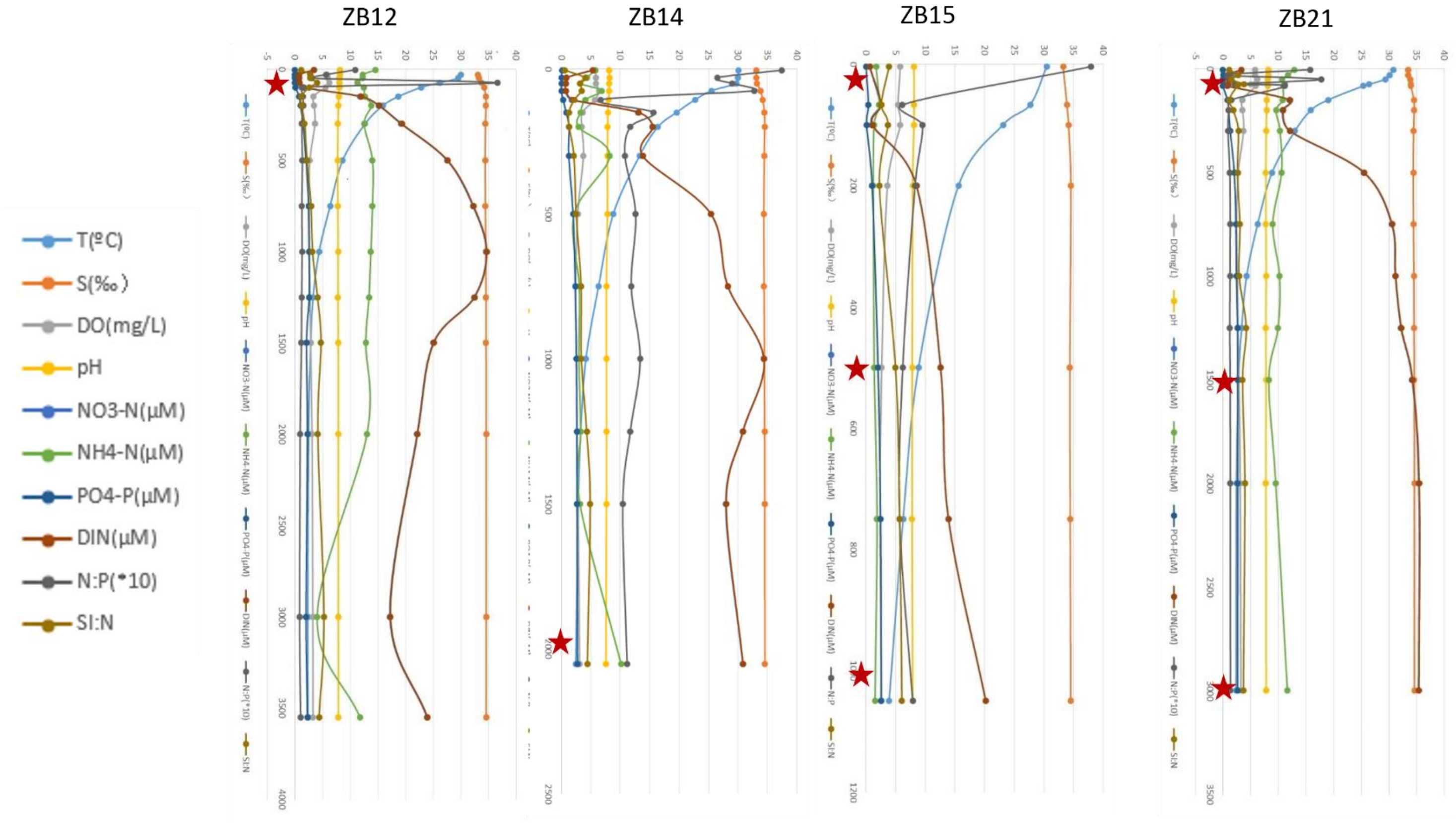

Fig S1. Physicochemical characteristics of the samples. The red star indicates the sample location for obtaining sequence data.

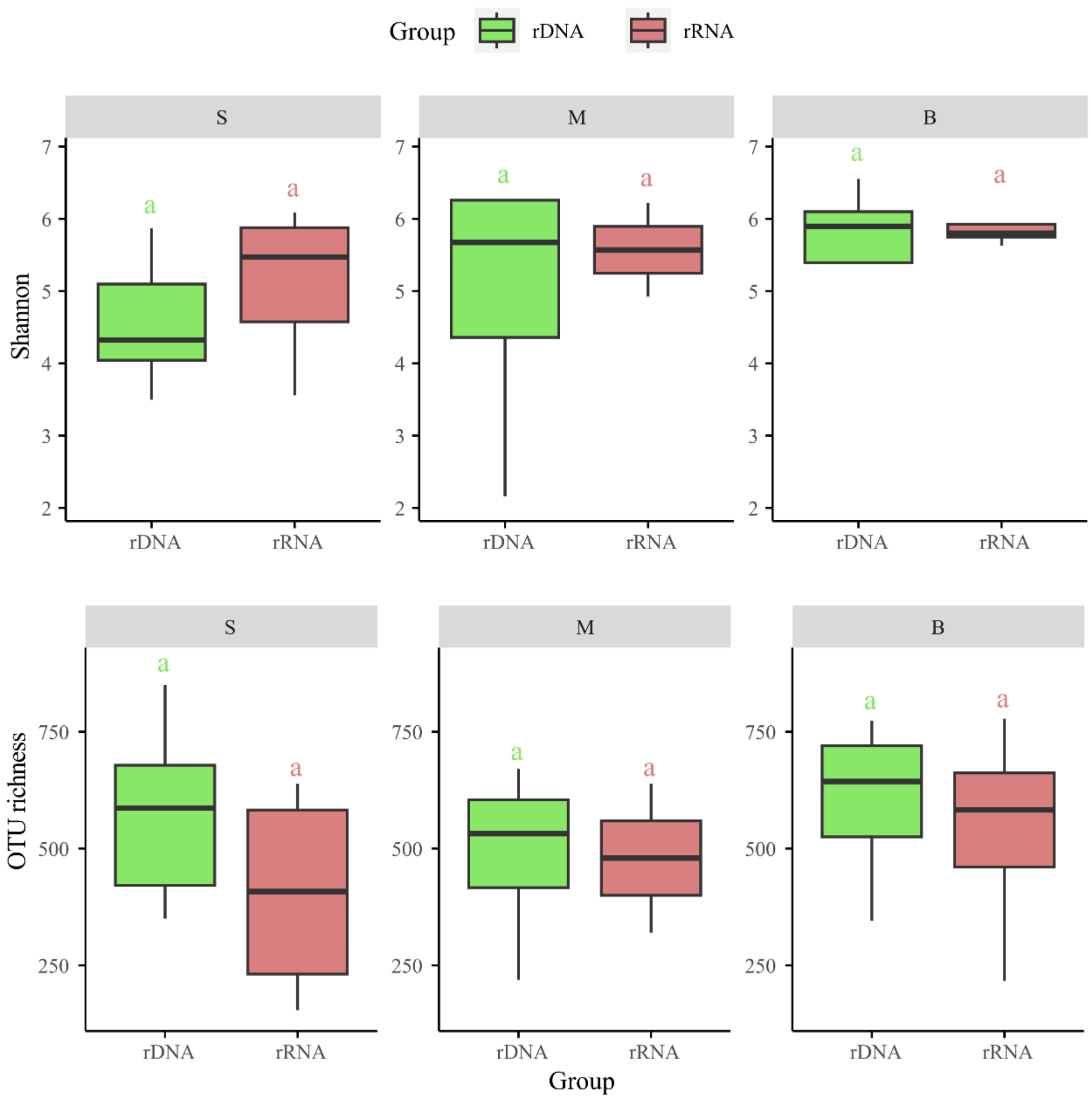

Fig S2. The alpha-diversity (Shannon indexes and OTUs richness) boxplot.

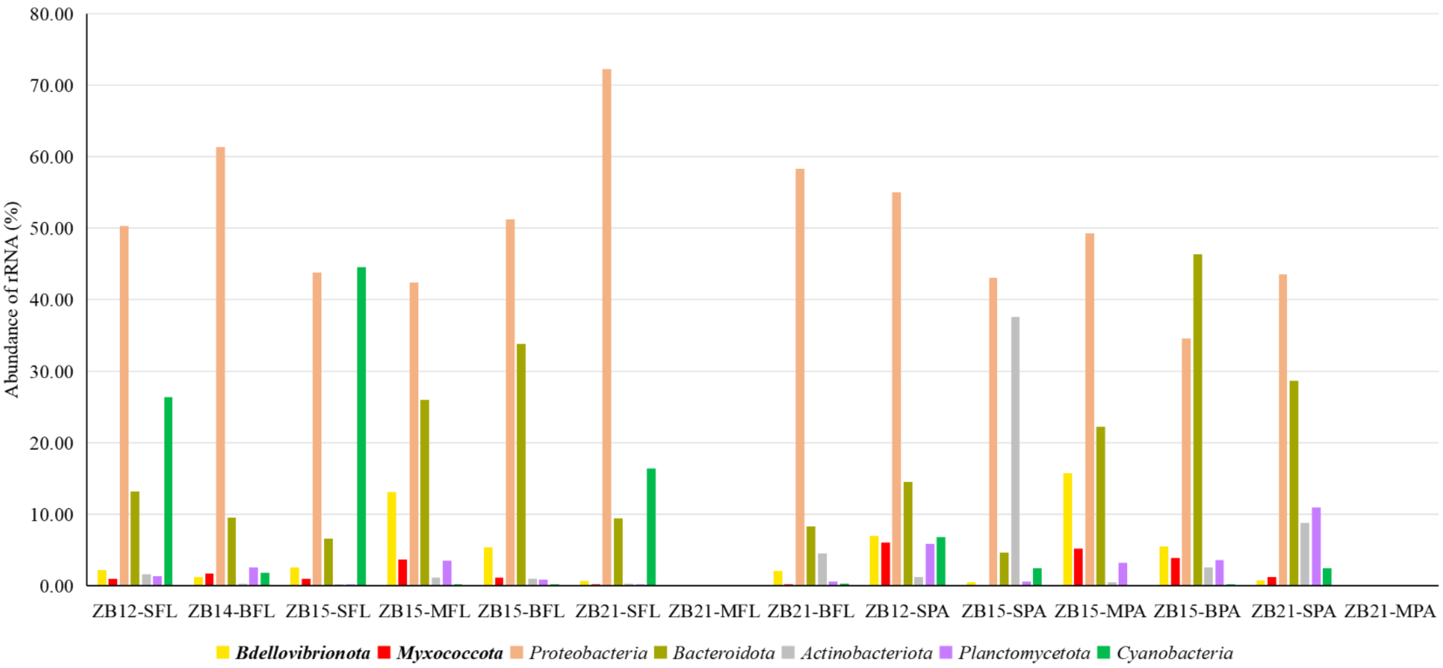

Fig S3. Abundance of rRNAs for major active taxa (>1%) in Zhenbei seamounts.

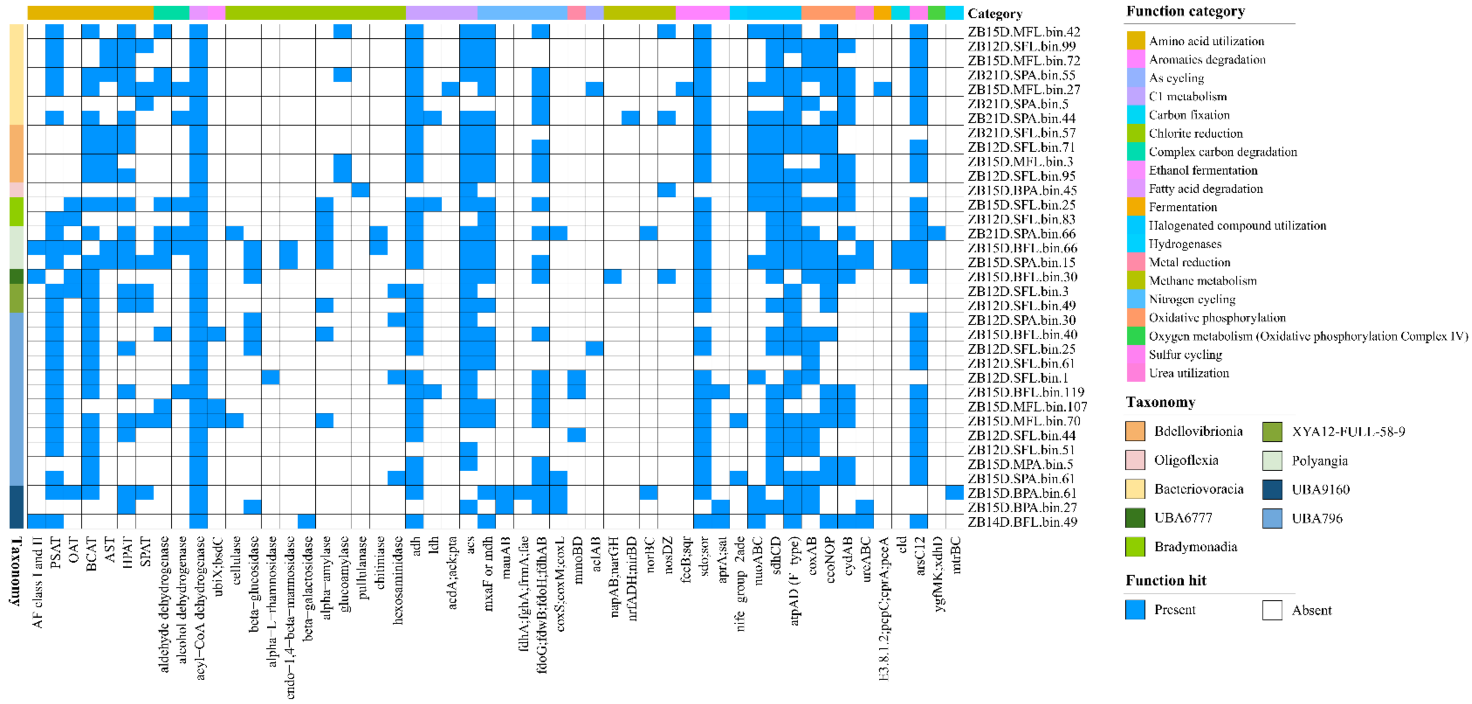

Fig S4. Metabolic and biogeochemical traits in the *Bdellovibrionotal* and *Myxococcotal* MAGs predicted with METABOLIC software.

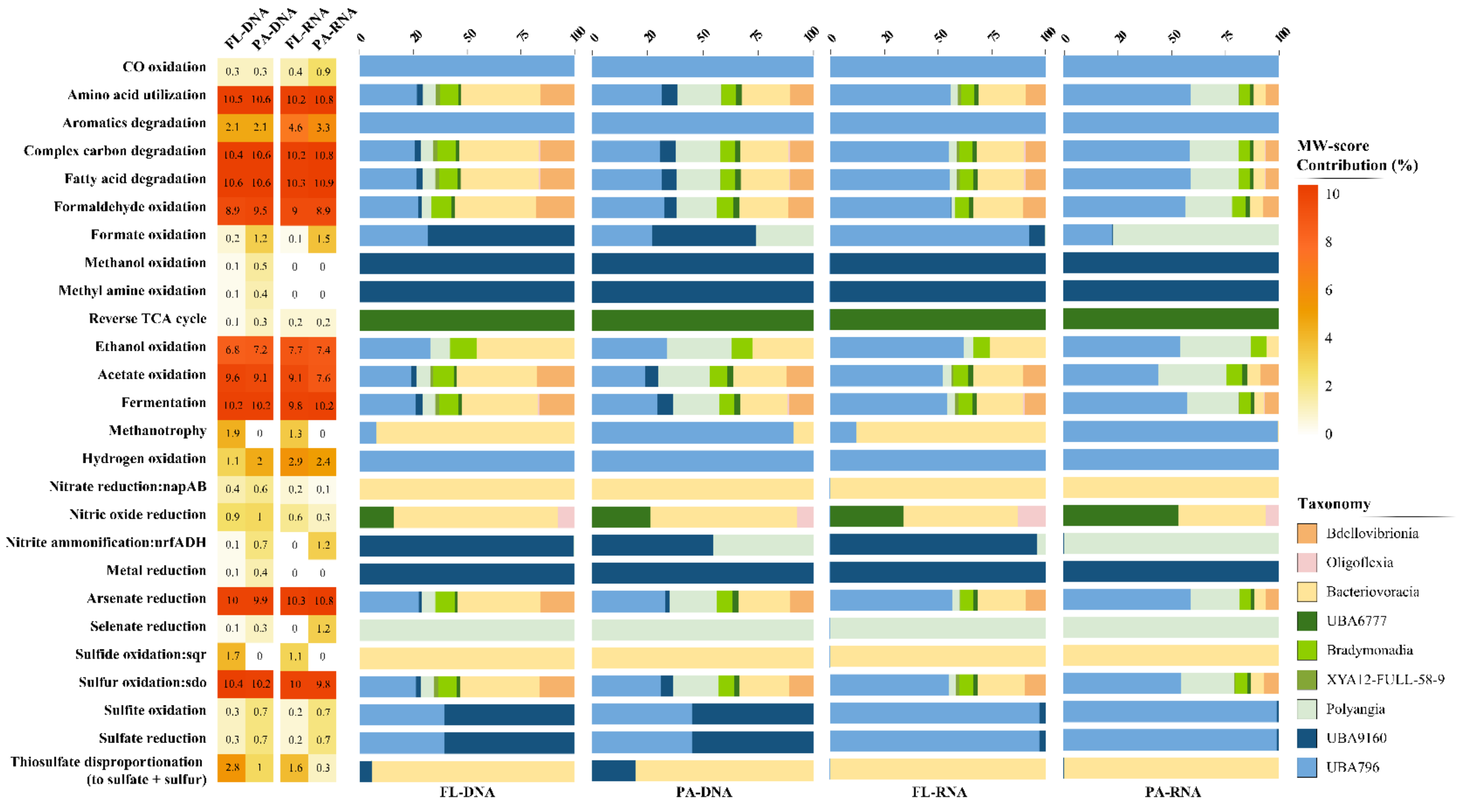

Fig S5. Community metabolism and the contribution of each order of predator bacteria based on the MW-scores (metabolic weight score). Heat maps represent the relative contribution of each function in metagenomic (DNA) and metatranscriptomic (RNA) samples. The bar charts show the taxonomic composition of each function in DNA and RNA samples.

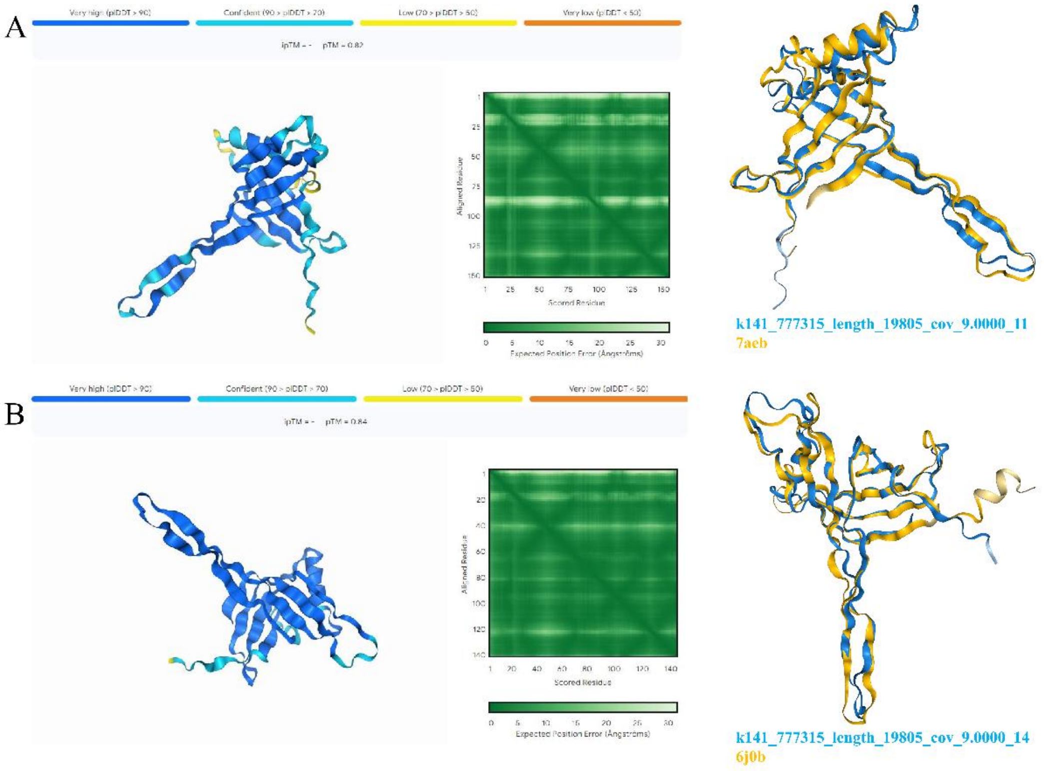

Fig S6. Structural prediction and comparison of phage T4 gp19 proteins encoded in ZB15-SPA-bin61. The structures of k141_777315_length_19805_cov_9.0000_11 (A) and k141_777315_length_19805_cov_9.0000_14 (B) are predicted and compared. The PDB numbers of the proteins, most similar in structure to the query proteins in the database, are highlighted in orange.

Supplementary Tables Legends

Table S1. The temperature, pH, and geochemistry data associated with each of the samples analyzed for this study.

Table S2. Samples with successful sequencing.

Table S3. The 16S rRNA and rDNA sequencing statistics of each sample analyzed for this study.

Table S4. The OTU table and representative sequences.

Table S5. The alpha diversity index of samples.

Table S6. The relative abundance and rRNA/rDNA of major bacterial phyla.

Table S7. Assessment of abundance and activity of each phylum based on its rDNA and rRNA/rDNA.

Table S8. Blast analysis of several specific OTUs of phyla *Bdellobibrionota* and *Myxococcota*.

Table S9. The reads number of trimmed metagenomic and metatranscriptomic data.

Table S10. The basic information of assembly results.

Table S11. *Bdellobibrionota* and *Myxococcota* MAGs retrieved from the assembled contigs.

Table S12. The taxonomy, quality, and relative abundance of *Bdellobibrionota* and *Myxococcota* MAGs.

Table S13. The top 10 genes with the high relative abundance expressed in each *Bdellobibrionota* or *Myxococcota* MAG.

Table S14. The highest relative abundance expressed genes in each *Bdellobibrionota* or *Myxococcota* MAG.

Table S15. The TPM for T4-like virus tail tube protein gp19 of *Myxococcota* MAGs.

Table S16. Blastp analysis of several PLTS-like cluster detected in *Myxococcota* MAGs.

Table S17. The number of CAZys observed in each *Bdellobibrionota* or *Myxococcota* MAG.

Table S18. The expression of GH involoved in the degradation of bacterial cell wall detected in *Bdellovibrionota* and *Myxococcota* MAG.

Table S19. Concentrations of DNA and RNA extracted from the membrane filters as determined by Qubit 3.0.
